# DEFECTIVE KERNEL1 regulates cellulose synthesis and affects primary cell wall mechanics

**DOI:** 10.3389/fpls.2023.1150202

**Published:** 2023-03-14

**Authors:** Lazar Novaković, Gleb E. Yakubov, Yingxuan Ma, Antony Bacic, Kerstin G. Blank, Arun Sampathkumar, Kim L. Johnson

**Affiliations:** ^1^ School of BioSciences, University of Melbourne, Parkville, VIC, Australia; ^2^ School of Biosciences, Max Planck Institute of Molecular Plant Physiology, Potsdam, Germany; ^3^ Faculty of Science, University of Nottingham, Leicestershire, United Kingdom; ^4^ La Trobe Institute for Sustainable Agriculture and Food, La Trobe University, Bundoora, VIC, Australia; ^5^ Mechano(bio)chemistry Department, Max Planck Institute of Colloids and Interfaces, Potsdam, Germany; ^6^ Institute of Experimental Physics, Johannes Kepler University, Linz, Austria

**Keywords:** DEFECTIVE KERNEL1, cellulose, cell wall, mechanical properties, cellulose synthase complex

## Abstract

The cell wall is one of the defining features of plants, controlling cell shape, regulating growth dynamics and hydraulic conductivity, as well as mediating plants interactions with both the external and internal environments. Here we report that a putative mechanosensitive Cys-protease DEFECTIVE KERNEL1 (DEK1) influences the mechanical properties of primary cell walls and regulation of cellulose synthesis. Our results indicate that DEK1 is an important regulator of cellulose synthesis in epidermal tissue of *Arabidopsis thaliana* cotyledons during early post-embryonic development. DEK1 is involved in regulation of cellulose synthase complexes (CSCs) by modifying their biosynthetic properties, possibly through interactions with various cellulose synthase regulatory proteins. Mechanical properties of the primary cell wall are altered in *DEK1* modulated lines with DEK1 affecting both cell wall stiffness and the thickness of the cellulose microfibril bundles in epidermal cell walls of cotyledons.

## Introduction

The polysaccharide rich extracellular matrix of plant cells has crucial roles in providing mechanical support, enabling water transport, cell-to-cell communication, and is a determining factor for turgor driven morphogenesis (Cosgrove, 2005). Cell walls vary in chemical composition and mechanical properties to support the different functional requirements of the cell. In primary walls of *Arabidopsis* the major components are cellulose, xyloglucan and pectins. Changes in cell wall composition and interaction between its component polymers greatly affect its mechanical properties and in turn impact growth and development of the plant (Zhang et al., 2019; Wang et al., 2020). Cellulose is a significant component of most types of cell walls, organized into cellulose microfibrils (CMF) that impart mechanical strength to the cell wall (Johnson et al., 2018; Zhang et al., 2019). Cellulose is synthesized by membrane bound CELLULOSE SYNTHASE (CESA) complexes (CSCs) organized in heterotrimers (Persson et al., 2007; Nixon et al., 2016; Polko and Kieber, 2019). Cellulose chains synthesized by CESAs form CMFs in the apoplast and become entangled in the cell wall matrix (Johnson et al., 2018). Mutants for either different CESA genes, such as *prc1-1*, or for regulatory proteins, such as *csi1/pom2* and *korrigan* (*kor*), have decreased cellulose content and growth defects (Fagard et al., 2000; Lane et al., 2001; Bringmann et al., 2012; Vain et al., 2014). Mutants for pectin and hemicellulose synthesis , such as either *quasimodo2* or *xyloglucan xylosyltransferase 1, 2* (*xxt1xxt2*) have less stiff cell walls than wild type (WT) plants. Lack of either pectin or xyloglucans (XGs) in these mutants decreases bundling of CMFs, changing the mechanical properties of cell walls and initiating complex signaling cascades that result in developmental defects (Xiao and Anderson, 2016; Du et al., 2020). Decreased synthesis of pectins and XGs also results in slower CSCs and less cellulose being produced, demonstrating that all components of the cell wall contribute to maintenance of its biochemical composition and integrity, in a continuous feedback loop-like manner (Xiao and Anderson, 2016; Du et al., 2020).

Proteins capable of sensing changes in biomechanical properties/mechanical signals at the level of the apoplast (PM/cell wall) are important for maintenance of cell wall integrity (CWI) (Engelsdorf and Hamann, 2014; Vaahtera et al., 2019). Previous studies have identified several protein families involved in CWI sensing and monitoring the mechanical status of the cell wall. These proteins belong to a family of *Catharanthus roseus* receptor like kinases (CrRLK), wall associated kinases (WAKs) and mechanosensitive (MS) ion channels (Vaahtera et al., 2019). Some of these proteins (eg. WAK2 and FERONIA (FER)) interact directly with pectins *via* their extracellular domains, while others (eg. MS ion channels), sense mechanical changes in the PM caused by mechanical perturbations of the cell wall. Changes in cell wall mechanics caused by either different developmental processes or environmental stress factors are sensed by these CWI sensors, eliciting different intra- and extra-cellular responses (Kohorn, 2009; Feng et al., 2018; Gjetting et al., 2020). Rapid mechanically activated (RMA) calcium currents are known to be activated by mechanical signaling. A functioning DEFECTIVE KERNEL1 (DEK1) protein has been shown to be crucial for RMA activity in *Arabidopsis* (Tran et al., 2017). DEK1 has been identified as a regulator of cell wall biochemical composition and structure (Johnson et al., 2008; Demko et al., 2014; Galletti et al., 2015; Amanda et al., 2016; Amanda et al., 2017).

DEK1 is a major regulator of plant development and growth (Johnson et al., 2005; Johnson et al., 2008). Loss-of-function mutants of *dek1* result in embryo lethal phenotypes in both *Arabidopsis* and maize (Becraft et al., 2002; Johnson et al., 2005). Plants with reduced levels of DEK1 such as *dek1* RNA interference and artificial microRNA (amiRNA) lines show a range of severe phenotypes, such as an almost complete absence and misspecification of the epidermal layer (Johnson et al., 2005), an irregular epidermal layer made of large cells (Johnson et al., 2008) or a loss of cell-to-cell adhesion and occasional gaps in the epidermal layer (Galletti et al., 2015). In addition, DEK1 indirectly controls HD-ZIP IV transcription factors which are involved in the regulation of epidermal identity (Galletti et al., 2015). It was proposed that DEK1 could act as a mediator of cellular adhesion by receiving and integrating signals from the apoplast and facilitating cell-to-cell contact through maintenance of epidermal identity (Galletti et al., 2015).

DEK1 belongs to the calpain superfamily of regulatory proteases. Calpains function in a range of cellular signaling pathways by modulating their targets through proteolytic processing resulting in altered protein activity, localization, substrate specificity or stability (Ono and Sorimachi, 2012). DEK1 is the only identified member of the calpain family in land plants (Lid et al., 2002; Demko et al., 2014) and its overall structure is distinct from the mostly cytosolic animal calpains (Lid et al., 2002; Wang et al., 2003). DEK1 is an approx. 240 kDa transmembrane protein with a complex structure including 21-23 transmembrane domains (TM), a loop domain, a cytoplasmic regulatory juxtamembrane domain and a proteolytically active CALPAIN domain close to its C-terminus (Lid et al., 2002; Johnson et al., 2008). DEK1 has been shown to undergo autocatalytic cleavage to release the CALPAIN domain into the cytoplasm where it is proposed to proteolytically cleave target proteins to alter their activity (Johnson et al., 2008; Amanda et al., 2016). The catalytic activity of DEK1 CALPAIN domain is dependent on calcium (Wang et al., 2003), as shown for animal calpains (García Díaz et al., 2006).Complementing *dek1* mutant lines of either *Arabidopsis* or *Physcomitrella patens* with only the CALPAIN domain is sufficient to rescue mutants, confirming the CALPAIN region as the catalytically active domain of the protein (Johnson et al., 2008; Demko et al., 2014). Over-expression of CALPAIN (*OE CALPAIN*) results in altered cell division and expansion, unlike over-expression of the full length DEK1, suggesting the TM and juxtamembrane regions of DEK1 act as regulatory domains (Johnson et al., 2008).

Studies performed on an over-expressor of CALPAIN (*OE CALPAIN*) and on *amiRNA DEK1* lines have shown that DEK1 is a major regulator of cell wall composition and structure in the epidermis of *Arabidopsis* leaves (Amanda et al., 2016). Immunohistochemical studies on *OE CALPAIN* lines showed a significant increase in antibody density for both cellulose, high- and low-methyl esterified homogalacturonan (HG) and arabinan while the same cell wall components were less abundant in epidermal cell walls of *amiRNA DEK1* compared with the WT (Amanda et al., 2016).

Increased labelling of epitopes for crystalline cellulose were specifically observed in epidermal cell walls of leaves and stems with no change in overall cellulose content in leaves of *OE CALPAIN-GFP* in a *dek1-3* mutant background and an *amiDEK1* line (Amanda et al., 2016). In this study, we investigated the role of DEK1 in the regulation of cellulose synthesis at a molecular level. An overexpressor of CALPAIN domain of DEK1 in WT background (pRPS5A:CALPAIN-6XHIS, *OE CALPAIN*, Galletti et al., 2015) and an EMS single point mutant, *dek1-4*, which has a C to T nucleotide substitution in the *CALPAIN* domain and has mild phenotypes (Roeder et al., 2012) were used in this study to enable more direct comparisons to WT plants. Results of this study demonstrate that DEK1 is involved in cellulose synthesis in *Arabidopsis* cotyledon pavement cells by regulating the dynamics of CSCs. Our data also indicate that DEK1 influences cell wall mechanics.

## Results

### DEK1 modulated lines show altered responses to isoxaben-induced cellulose perturbations

**Figure 1:**
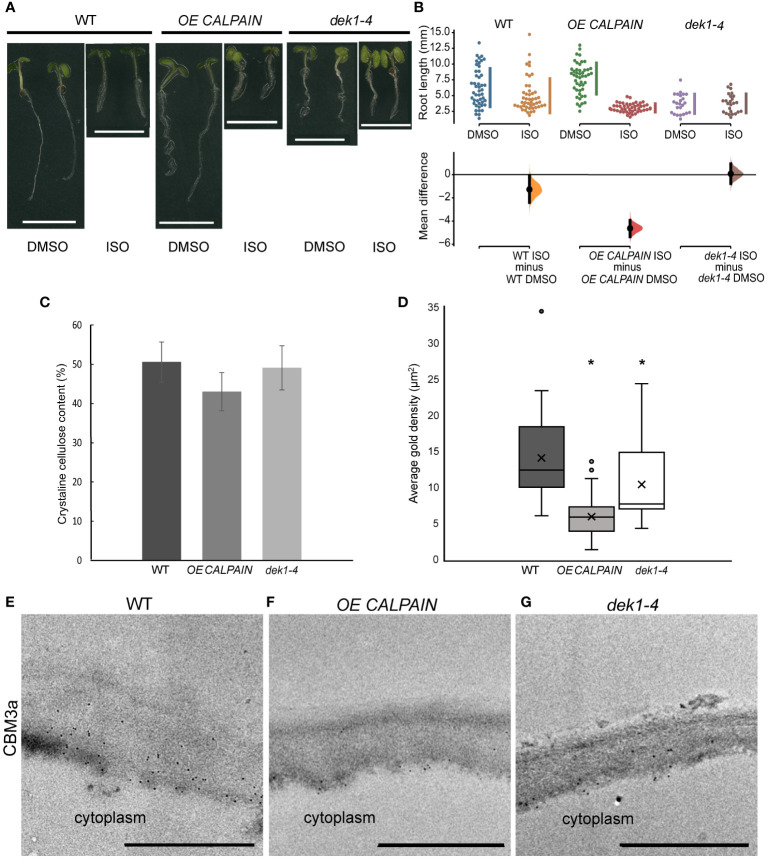
Isoxaben treatment and cell wall analysis of WT and *DEK1* modulated *Arabidopsis* lines. **(A)** Representative images of 6-day old WT, *OE CALPAIN* and *dek1-4* seedlings grown on media with either DMSO (control) or 2 nM isoxaben (ISO), scale bar = 5 mm. **(B)** Multiple two-group estimation analysis of root length for each genotype between plants grown on media with DMSO and isoxaben. The mean differences are shown in the Cumming estimation plot (lower panel). The raw data are plotted on the upper axes; each mean difference is plotted on the lower axes as a bootstrap sampling distribution. Mean differences are depicted as dots; 95% confidence intervals are indicated by the ends of the vertical error bars. The difference between ISO/DMSO plants was WT -2.7 mm (95% CI-2.39, -0.0585, p = 0.0332), *OE CALPAIN* -4.63 mm (95% CI -5.35, -3.95, p = <0.0001) and *dek1-4* 0.0824 mm (95% CI -0.762, 0.94, p = 0.848). N=47 WT DMSO, WT ISO, *OE CALPAIN* DMSO, 50 *OE CALPAIN* ISO, 24 *dek1-4* DMSO, 25 *dek1-4* ISO **(C)** Crystalline cellulose contents of AIR cell wall preparations of 10-day old seedlings. Analysis performed on two biological replicates and two technical replicates. Error bars are representing standard error. No statistical significance observed p>0.05, Students t-test. **(D)** Average gold particle density (indicated by 'x') for CBM3a labelled cellulose in outer periclinal epidermal walls was WT 14.09 ±1.01 µm^-2^, *OE CALPAIN* 5.93±1.01 µm^-2^ and *dek1-4* 10.41±1.44 µm^-2^. Asterisk represents statistical significance, p<0.0001 for *OE CALPAIN*, p<0.05 for *dek1-4*, unpaired Student t-test. n=36 WT, 25 *OE CALPAIN* and 16 *dek1-4* epidermal cell walls. Analysis performed on biological duplicates. **(E–G)** Transmission electron micrographs of outer epidermal cell walls of 10-day old cotyledons showing immunogold labelled CBM3a cellulose binding. Scale bars = 500 nm.

To investigate the possible involvement of DEK1 in regulation of cellulose biosynthesis, WT and *DEK1* modulated lines were treated with isoxaben. Isoxaben is a plant-specific herbicide which inhibits the synthesis of cellulose through interacting with CESAs and causing them to be removed from the PM and internalized into the cell (Heim et al., 1990; Shim et al., 2018). Isoxaben-induced disruption of cellulose synthesis causes reduced root growth and cell swelling. Cellulose deficient mutants have been shown to display altered responses to isoxaben treatment (Engelsdorf and Hamann, 2014; Vaahtera et al., 2019). We measured root lengths of 6-day old WT and *DEK1* modulated plants grown on media supplemented with either 2 nM isoxaben or DMSO as a control (**Figure 1A**). In response to isoxaben treatment WT plants showed a significant reduction of root length (**Figure 1B**). For DMSO control plants no differences in root lengths between WT and *OE CALPAIN* seedlings were observed. In contrast, we observed significant differences between the mean root length of WT and *OE CALPAIN* when grown on isoxaben, shown by the 95% CIs of the mean differences of these two groups not overlapping (**Figure 1B**). Estimation statistics analysis showed high mean differences in mean root length between *OE CALPAIN* plants grown on 2 nM isoxaben and the DMSO controls (**Figure 1B**). In *dek1-4* mutants grown on DMSO media the mean difference in root length compared to WT indicated significantly shorter root growth (**Figure 1A**). In response to isoxaben-induced mechanical perturbation of the cell wall *dek1-4* mutants resulted in no change in root growth compared to controls (**Figures 1A, B**). In addition, no significant differences in the mean difference were observed between *dek1-4* and WT (**Figure 1B**, 95% CI-s are overlapping). Changes in cellulose levels are known to influence plant tolerance to abiotic stresses such as salt (Endler et al., 2015). The response of *DEK1* modulated lines to salt treatment was investigated. WT showed significantly reduced root growth after transfer to salt treatment compared to plants transferred to control media (**Supplementary Figure 1**). The response of *OE CALPAIN* plants to salt was more moderate with a smaller reduction in root growth than WT (**Supplementary Figure 1**). A similar insensitive response to that of isoxaben treatment was observed for *dek1-4*, with a small mean difference between root length in control and salt-grown plants (**Supplementary Figure 1**).

### DEK1 is involved in fine tuning of epidermal cell wall composition

Studies of *DEK1* modulated lines grown on isoxaben and salt suggest cellulose content and/or assembly is altered. Cell wall linkage analysis was undertaken on alcohol insoluble residue (AIR) wall preparations to investigate potential wall compositional differences in 10-day old seedlings of *DEK1* modulated lines compared to WT. No statistically significant differences were found (**Supplementary Figure 2**; **Supplementary Table 1**). A trend towards reduced levels of cellulose in *OE CALPAIN* lines was observed and this was investigated further using the Updegraff (1969) method which determines crystalline cellulose content. Of the AIR, crystalline cellulose constituted 51% (w/w) in WT, 42% in *OE CALPAIN* and 48% in *dek1-4* (**Figure 1C**). Previous studies have shown DEK1-related changes in cellulose and pectin content predominantly occurs in the outer epidermal cell walls of both young and mature leaves and inflorescence stems (Amanda et al., 2016; Amanda et al., 2017).

Immunofluorescence and immuno-gold labelling of cell wall epitopes was used to determine if wall composition differs at the tissue/cellular scale that may be beyond the resolution of the whole seedling chemical analyses. CBM3a labelling of cellulose epitopes showed slightly lower fluorescence intensity in the outer periclinal epidermal cell wall of both *DEK1* modulated lines compared to WT (**Supplementary Figures 3A–C**). TEM imaging of the cotyledon epidermal cell walls showed significantly decreased immuno-gold labelling of cellulose epitopes with CBM3a in both *DEK1* modulated lines compared with WT (**Figures 1D–G**). Cotyledon sections labelled with Calcofluor white to detect cellulose, JIM5 and JIM7 to detect partially and highly methyl-esterified HG pectin, respectively, and LM15 to detect XG revealed no obvious differences in fluorescence signal between WT and *DEK1* modulated lines (**Supplementary Figure 4**). We performed immuno-gold labelling with JIM5 and JIM7 antibodies that detect HG pectins in cotyledon epidermal cell walls (**Supplementary Figure 5**). Our results show that *dek1-4* had significantly higher average density of JIM5 immuno-gold particles compared with WT, while *OE CALPAIN* has a significantly lower count of JIM5 immuno-gold particles (**Supplementary Figures 5A–C, G**). Levels of partially-methyl esterified HG detected by the JIM7 antibody were significantly higher in *OE CALPAIN* and significantly lower in *dek1-4* compared to WT (**Supplementary Figures 5D–F, H**).

### CESA transcript levels show no differences between WT and *DEK1* modulated lines

Isoxaben growth assays, immuno-labelling and cell wall compositional analysis suggest DEK1 influences levels of cellulose, particularly in epidermal walls. To determine how changes in cellulose may have come about we investigated CSCs at both the transcriptional and post-transcriptional levels. Previous studies have shown that modulation of DEK1 affects expression of numerous cell wall-related genes, however *CESA* levels were unaffected (Johnson et al., 2008; Amanda et al., 2016). *CESA* transcript levels were investigated in 6-day old seedlings of WT, *OE CALPAIN* and *dek1-4* lines using qRT-PCR. No significant differences in transcript levels of *CESA1*, *CESA3* and *CESA6*, encoding primary wall CESAs, were observed between WT and *DEK1* modulated lines (*p*>0.05) using fold change analysis (**Supplementary Figure 6**). These data suggest DEK1 influences cellulose biosynthesis at the post-transcriptional level.

### *DEK1* modulated lines influence mobility and not density of CSCs

**Figure 2:**
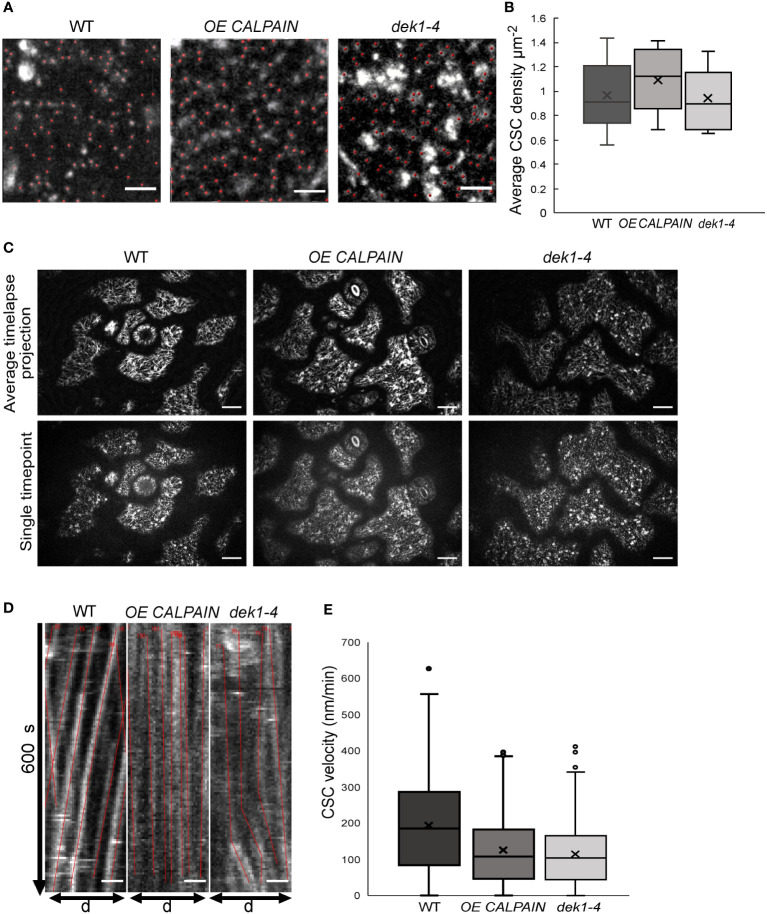
Analysis of cellulose synthase complex (CSC) migration at the plasma membrane in WT and *DEK1* modulated *Arabidopsis* lines. **(A)** Representative micrographs of 3-day old *Arabidopsis* cotyledon epidermal pavement cells' membrane patches expressing GFP.CESA3 fluorescent marker used for CSC (red dots) density calculation in Col-0 and *DEK1* modulated Iines, Scale bars = 2 µm, **(B)** Box plots showing average density of CESA particles in 3-days old cotyledon pavement cells. WT 0.89 ± 0.04 µm^-2^, *OE CALPAIN* 0.93 ± 0.04 µm^-2^ and *dek1-4* 0.82 ± 0.04 µm^-2^, N=6 plants for WT, 31 cells; 6 plants for *OE CALPAIN*, 33 cells; 6 plants for *dek1-4*, 27 cells. No statistical significance observed between WT and *DEK1* modulated lines, p=0.5647 for *OE CALPAIN*, p=0.2705 for *dek1-4*. **(C)** Representative images of 3 day old cytoledon epidermal pavement cells used for generation of kymographs. Upper panel represents average projections of time lapses, bottom panel represents a single frame of a corresponding time lapse, N=6 plants per genotype. Scale bars=10 µm. **(D)** Representative kymographs showing migration of CESA complexes in 3-day old cotyledon pavement cells of Col-0 and *DEK1* modulated lines. Red lines represent trajectories of individual CESA particles. WT, N=6 plants, 937 CSC particles tracked; *OE CALPAIN*, N=6 plants, 982 CSC particles tracked; *dek1-4*, N-6 plants, 692 CSC particles tracked. Analysis performed on biological duplicates. Scale bars=10 µm. **(E)** Box plots of CESA velocities obtained from kymographs WT 192.41 ± 4.16 nm min^-1^, OE CALPAIN 124.11 ± 3.01 nm min^-1^ and *dek1-4* 113.91 ± 3.09 nm min^-1^. Asterisk represent statistical significance Student (p<0.0001, Student t-test).

Changes in cellulose content could be explained by differences in the number of CSCs at the PM. *OE CALPAIN* and *dek1-4* lines were crossed with a pCESA3:GFP : CESA3 mCh : TUA5 (GFP : CESA3) reporter line and the number of GFP : CESA3 complexes on pavement cell PM patches quantified (**Figure 2A**). No statistically significant differences were detected in CSC particle density between WT and *DEK1* modulated lines (**Figure 2B**). We then proceeded to investigate if DEK1 could affect the velocity of the CSC. Investigation of CSC velocity is commonly used as a proxy for determining cellulose synthesis rates at the cellular level. In brief, the more cellulose CESAs synthesize, the faster CSCs will move along within the plane of the PM (Paredez et al., 2006; Diotallevi and Mulder, 2007; Fujita et al., 2011). CSCs at the PM of the periclinal epidermal cells of 3-day old cotyledons were tracked during 10-min time lapses (**Figure 2C**) and kymographs of GFP-labelled CESA3 particles (**Figure 2D**) were used to obtain velocities of CSCs (**Figure 2E**). CSC velocities in both *OE CALPAIN* and *dek1-4* mutant lines were significantly reduced compared to WT (**Figure 2E**). A reduction of CSC velocities in the periclinal cell walls of cotyledon pavement cells is consistent with the findings of immuno-histochemical assays that indicate significantly reduced cellulose levels in *OE CALPAIN* compared to WT (**Figure 1D**).

### CSC membrane trafficking is significantly altered in *DEK1* modulated lines

**Figure 3:**
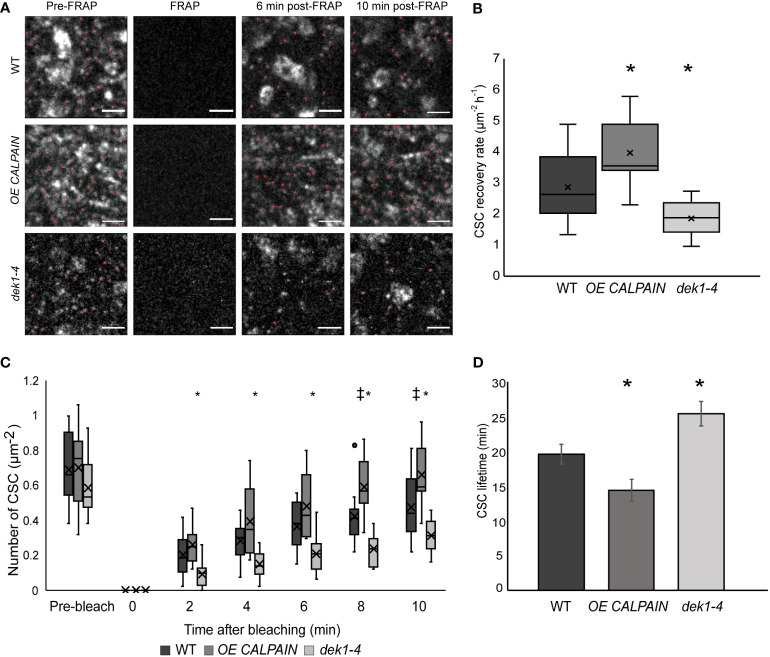
Delivery and resident time of CESAs at the plasma membrane of WT and *DEK1* modulated lines in *Arabidopsis* using fluorescent-recovery after photo-bleaching (FRAP). **(A)** Representative images showing delivery of new CESA complexes (red dots) after photo-bleaching in 3-day old cotyledon pavement cells of WT, *OE CALPAIN*, *dek1-4*. Scale bars = 2 µm. **(B)** Box plots showing average recovery rate of CESA particles following FRAP in cotyledon pavement cells WT 2.86 ± 0.25 CSC µm^-2^h^-1^ , *OE CALPAIN* 3.96 ± 0.36. CSC µm^-2^h^-1^, *dek1-4* 1.87 ± 0.15 CSC µm^-2^h^-1^. Asterisk represent statistical significance (p<0.0188 for *OE CALPAIN*, p<0.0033 for *dek1-4*, Student's t-test ). **(C)** Box plots representing recovery of CESA particles after photo-bleaching at each time-point. Symbols above box plots represent statistical significance (p=0.0102 and 0.0188 for *OE CALPAIN*, p=0.0001 and -0.0055 for *dek1-4*, Student's t-test). **(D)** Histogram representing average lifetime of CESA complexes at the PM, WT 19.56 ± 1.42 min, *OE CALPAIN* 14.33 ± 4.69 min and *dek1-4* 25.36 ± 1.73 min. Asterisk above boxplot represents statistical significance (p<0.05, Student's t-test), error bars represent standard error. **(B–D)** Analysis was performed in two biological replicates for WT and *dek1-4*, and one replicate for *OE CALPAIN*. N = 7 cotyledons for WT, 3 cotyledons for *OE CALPAIN*, 6 cotyledons for *dek1-4*;N =16 cells for WT, 9 cells for *OE CALPAIN*, 14 cells for *dek1-4*.

To investigate if DEK1 might be regulating other functional properties of CSC complexes, delivery rate of CSCs and their lifetime at the PM was examined. Fluorescence recovery after photobleaching (FRAP) experiments were performed on *OE CALPAIN* and *dek1-4* plants crossed into a GFP : CESA3 reporter line to determine if the rate of exocytosis of CSCs into the PM was altered. Imaging of GFP : CESA3 particles during the 10 min following photobleaching (**Figure 3A**) showed CESAs in *OE CALPAIN* had significantly faster recovery after FRAP than WT and *dek1-4* had significantly slower recovery (**Figures 3B, C**). Studies of the lifetime of CSC particles at the PM showed the opposite trends (**Figure 3D**). *OE CALPAIN* CSCs spent significantly shorter time in the PM than WT CSCs, while *dek1-4* CSCs stayed significantly longer in the PM before undergoing endocytosis (**Figure 3D**).

### DEK1 affects cell wall mechanics of cotyledon pavement cells

The defects in CESA dynamics observed in cotyledon pavement cells prompted us to examine the possible role of DEK1 in influencing structure and physical properties of the CMFs and consequently the mechanical properties of the cell wall. We used atomic force microscopy (AFM) to image and mechanically characterize native cell walls.

**Figure 4:**
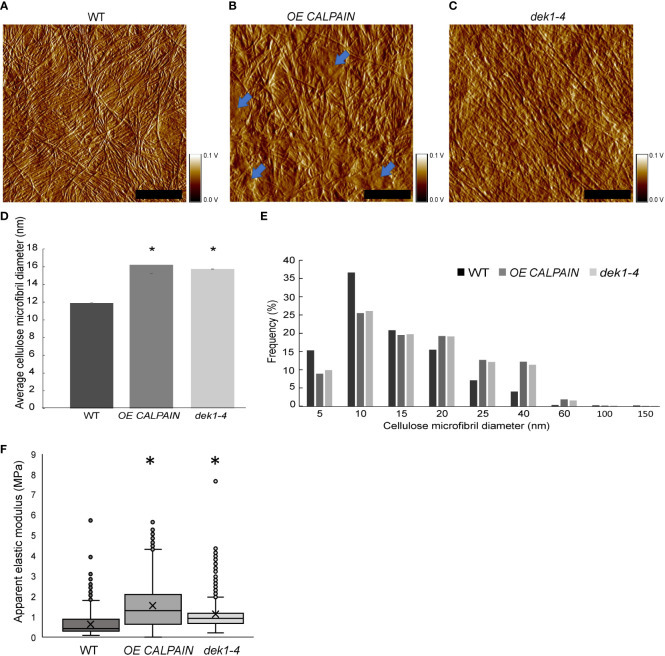
AFM imaging and and nanomechanical characterization of outer periclinal cell walls of *Arabidopsis*. **(A–C)** AFM amplitude channel images of the inner face (juxtaposed to the PM) of periclinal primary cell walls in abaxial cotyledon pavement cells of 10-days old seedlings of WT, *OE CALPAIN and dek1-4.* Samples were imaged using tapping mode in air. Arrows indicate amorphous cell wall regions. Scale bars=250 nm. Corresponding height channel AFM images are shown in **Supplementary Figure 8**. **(D)** Histograms representing average thickness of cellulose microfibrils in cotyledon epidermal cell walls, WT 11.09 ± 0.03 nm, *OE CALPAIN* 16.21 ± 0.03 nm and dek1-4 15.74 ± 0.03 nm. Asterisk represents statistical significance, p<0.0001, unpaired Student t-test. N=5 cotyledons, 10 cells, 1-2 regions per cell for WT; N=6 plants, 6 cells, 1-2 regions per cell for *OE CALPAIN*; N=4 cotyledons, 7 cells, 1-3 regions per cell for *dek1-4*. **(E)** Distribution of CMF thickness and frequency in WT, *OE CALPAIN* and *dek1-4*. **(F)** Box plots representing distribution of cell wall apparent elastic moduli generated after nanoindentation of extracted cotyledon epidermal cell walls in a direction perpendicular to CMFs of 10-day old cotyledons of WT 0.71 ± 0.01 MPa, *OE CALPAIN* 1.52 ± 0.03 MPa and *dek1-4* 1.11 ± 0.02 MPa. N=12 indented cotyledons from 12 different plants for WT (1427 indentation points), 9 cotyledons from 9 different plants for *OE CALPAIN* (1553 indentation points), and 12 cotyledons from 12 different plants for *dek1-4* (1509 indentation points). One cell per one cotyledon was indented in all genotypes. Asterisk represents statistical significance, p<0.0001, Student t-test. **(A–F)** All experiments were performed in two biological replicates.

We performed AFM imaging on dry cell wall monolayers of isolated abaxial epidermal cell walls from cotyledons of 10-day old *Arabidopsis* seedlings (**Figures 4A–C**; **Supplementary Figures 7, 8**). Imaging was done on the inner surface of the abaxial epidermis outer periclinal walls, which is the newest deposited layer of the cell wall, abutting the PM (**Supplementary Figure 7**). Cell wall images of WT clearly show the CMF network (**Figures 4A–C**; **Supplementary Figure 9**), as reported for onion epidermal cell wall monolayers imaged by AFM (Kafle et al., 2014; Zhang et al., 2016). Differences in the thickness of CMFs were observed in *OE CALPAIN* and *dek1-4* (**Figures 4A–C**) compared to WT. In *OE CALPAIN*, thicker bundles of CMFs were observed as well as amorphous deposits on the cell wall surface (**Figure 4B**). Diameters of the CMF bundles were quantified from height channel images (**Supplementary Figure 9**). Results showed that the CMF bundles of both *OE CALPAIN* and *dek1-4* had significantly higher mean diameters than WT CMFs (**Figure 4D**). Analysis of the distribution of CMF dimeters shows that both *DEK1* modulated lines have a higher frequency of bundled CMFs in the range of 15-40 nm whereas WT CMFs are mostly represented in the range of 1-15 nm, implying that WT has either an abundance of single CMFs (~3.5 nm is the diameter of a typical CMF (Zhang et al., 2016)) or smaller bundles of CMFs (**Figure 4E**).

Previous studies have shown that changes of cell wall composition in cell wall mutants of *Arabidopsis* can affect mechanical behavior of the cell wall and CMF properties (Xiao and Anderson, 2016). To examine cell wall mechanical properties in *DEK1* modulated lines, we performed AFM nanoindentation on the isolated abaxial epidermal cell walls of 10-day old *Arabidopsis* cotyledons. Nanoindentation generated force-displacement curves, were used to calculate cell wall elastic moduli (**Supplementary Figures 10A, C, E**). It is worth noting that within all analyzed genotypes, a large range of elastic moduli was observed (**Figure 4F**; **Supplementary Figures 10B, D, F**). In WT, values ranged from 0.1 Mpa to 5 Mpa, which is consistent with previous data showing highly heterogenous distribution of cell wall mechanical properties (Yakubov et al., 2016). Analysis of the apparent elastic modulus in *OE CALPAIN* and *dek1-4* suggests that both lines had significantly stiffer cell walls compared to WT (**Figure 4F**). These results suggest DEK1 is involved in regulating and maintaining mechanical properties of the cell wall.

## Discussion

In this study we show that DEK1 affects the synthesis of both cellulose and pectins in early developmental stages of *Arabidopsis*. Both *DEK1* modulated lines show decreased crystalline cellulose in outer epidermal cell wall of cotyledons. Velocity of CSCs in the epidermal pavement cell PMs, which is used as a proxy for the biosynthetic activity, is significantly reduced in both *DEK1* modulated lines, while the density of CSC complexes is similar to that of the WT. PM dynamics of CSCs is significantly altered in both lines, with *OE CALPAIN* CSCs having significantly slower CSC exocytosis and faster endocytosis, while these features of CSC dynamics were reversed in *dek1-4* plants. Epidermal cell wall stiffness was significantly increased in both *DEK1* modulated lines compared to WT.

### DEK1 in cellulose assembly

DEK1 is proposed to act as a mechano-sensor protein at the PM (Amanda et al., 2016; Tran et al., 2017). Upon perception of mechanical stimuli, originating either from other growing cells in the tissue or from a cell’s own turgor pressure, or an external environmental signal, the CALPAIN domain of DEK1 is proposed to be auto-catalytically released into the cytoplasm (Amanda et al., 2016; Tran et al., 2017). DEK1 CALPAIN likely acts as a regulatory protease (Wang et al., 2003; Johnson et al., 2008) and downstream signaling initiates changes in cell wall biosynthesis/remodeling and epidermal specification (Demko et al., 2014; Galletti et al., 2015; Amanda et al., 2016; Amanda et al., 2017). In this study we show DEK1 influences CESA dynamics, cellulose content, pectin re-structuring and mechanical properties of epidermal walls.

It is currently understood that slower movement of CSCs across the PM, a proxy for a lower rate of cellulose biosynthesis, results in less cellulose synthesized (Diotallevi and Mulder, 2007). The significant reduction of CSC velocity in *OE CALPAIN* and *dek1-4* (**Figures 2D, E**) support a role for DEK1 in regulation of CESA dynamics. Mutants for *CESA6/prc1-1*, and *CESA1*/*any1*, have significantly reduced CESA velocities in dark grown hypocotyls (Bischoff et al., 2011) that is proposed to be the major cause of a 30% reduction in growth (Fagard et al., 2000). There is no data in the literature that CESA proteins undergo proteolytic processing after insertion (by exocytosis) into the PM. RT-qPCR analysis of *CESA1,3* and *6* expression levels, and measurement of CESA density at the cotyledon pavement epidermal cell PMs showed no difference between WT and *DEK1* modulated lines. These data support DEK1 regulation of CSCs at the post-translational level. An interesting possibility is that DEK1 regulates CSC activity indirectly, potentially through interactions with either CESA regulatory proteins or the cytoskeleton components that guide CSCs. Candidates would include CSI1/POM2 that facilitate binding between CSCs and CMTs (Gu et al., 2010; Bringmann et al., 2012), the endo-1,4-beta-glucanase KORRIGAN (KOR) involved in CESA regulation (His et al., 2001; Vain et al., 2014) and PATROL1 (PTL1) that interacts with exocyst complex proteins and CS1/POM2 to deliver CSCs to the PM (Zhu et al., 2018). Mutants *kor1-1* and *kor1-3* display a similar reduction in CSC mobility to *DEK1* modulated lines when compared to WT plants (Vain et al., 2014). In addition *kor* mutants have been shown to have decreased cellulose content, but also increased pectin abundance, which was interpreted as a compensatory mechanism and part of feedback loops connecting cellulose and pectin synthesis to changes in the mechanical properties of the cell wall (His et al., 2001).

It could be conceivable that DEK1 is involved in CSC guidance along CMTs, either by modifying the activity and properties of CSI1/POM2 or some other CESA-CMT interface proteins. Reduced CSCs velocities and cellulose content is observed in *pom2-1* mutants compared to WT plants as well as spiral twisting of the entire rosette and leaves. Previous studies of lines with reduced levels/activity of *DEK1*, including *dek1-4*, showed epinastic curling of cotyledons and leaves (Roeder et al., 2012; Galletti et al., 2015). CMTs are responsive to mechanical stress and assumed to align in the direction of maximal stress (Hamant et al., 2008). During salt stress, CMTs are rapidly disassembled, and CSCs removed from the PM (Endler et al., 2015). COMPANION OF CELLULOSE SYNTHASE (CC) proteins bind to CMTs and promote salt tolerance by stabilizing CMTs and enabling cellulose synthesis. CMTs were shown to re-organize normally in *dek1-4* mutants in response to ablation in cotyledons, however, were more highly orientated than in WT pavement cells due to changes in cell shape (Galletti et al., 2015). A study in animals has shown that a non-proteolytic variant of CALPAIN, CALPAIN6 (CAPN6), stabilizes microtubules by binding to them and preventing their de-polymerization (Tonami et al., 2011). Unlike CAPN6, DEK1 CALPAIN has proteolytic activity, and it would be interesting to investigate if DEK1 CALPAIN can interact with CMT and potentially disrupt binding of CESA complexes (Tonami et al., 2011).

Upregulation of the exocytotic module of the CSC trafficking machinery in *OE CALPAIN* (**Figures 3B, C**) could be one explanation for the faster exocytosis of the CESAs, as observed by faster post-FRAP recovery of CSC particles (**Figures 3A, B**). Exocytosis of CSCs is regulated by a large protein complex (Polko and Kieber, 2019), which interacts with CSI1 and PTL1 to deliver CSCs to the PM (Zhu et al., 2018). The frequency of CSC insertions and alignment with the CMT was highly decreased and the delivery rate of CSC severely diminished in *csi1-3*/*ptl1-2* double mutants. In addition, *ptl* mutants also have lower CSC velocities, and both *ptl1-2* and *csi1-3*/*ptl1-2* lines have significant decreases in cellulose content (Zhu et al., 2018). The contrasting effect which *OE CALPAIN* and *dek1-4* have on CSC PM trafficking could mean that DEK1 is interacting with several components involved in CSC trafficking to (and in) the PM, and that its role in signaling involves multiple target proteins.

### DEK1 and cell wall mechanics

The epidermis is a key regulator of plant growth and this is influenced by the mechanical properties of the cell wall (Galletti et al., 2016; Moulia et al., 2021). In *DEK1* modulated lines, pectins and cellulose are altered yet the dynamics of these changes are unclear and if direct or indirect. Previous studies (Amanda et al., 2016; Amanda et al., 2017), have shown that DEK1 affects cell wall composition almost exclusively on the level of leaf outer epidermal cell walls. In leaf 4 of 17 day old *Arabidopsis* plants, increased labelling of CBM3a recognizing cellulose and JIM5 detecting low-methyl esterified HG was found in *dek1-3 OE CALPAIN GFP* compared to WT and an *amiRNA DEK1* line. In this study, decreased levels of CBM3a and JIM5 labelling was found in epidermal walls of cotyledons of *OE CALPAIN*. DEK1 potentially acts in different regulatory networks and/or on tissue specific targets during early and late developmental stages of *Arabidopsis*. Alternatively, changes in cell walls at later stages could be indirect and result from compensatory changes initiated by cell wall integrity pathways (Vaahtera et al., 2019). Further analysis is required to uncover the dynamics of DEK1-regulated wall changes in future.

Compensatory mechanisms to counteract changes in cell wall composition and biomechanics are known to occur, for example, pectins have been shown to increase in response to reduced levels in cellulose and lead to increased stiffness of the cell wall (His et al., 2001). DEK1 has previously been shown to regulate expression of pectin-related genes, including *GAUTs* and pectin methyltransferases as well as altered deposition of pectic polysaccharides in leaf epidermal cell walls of *OE CALPAIN GFP* (Amanda et al., 2016; Amanda et al., 2017). Mutants in pectin methyltransferase, *quasimodo2* and *tumorous shoot development2*, have decreased levels of both HGs and cellulose, decreased CESA velocities, thinner and more fragmented CMFs (Mouille et al., 2007; Du et al., 2020), while mutants in putative galacturonic acid transferase, *quasimodo1*, experience severe defects in cell-to-cell adhesion due to low HG-content (Bouton et al., 2002; Verger et al., 2018). Increased partially methyl-esterified HG in *dek1-4* is consistent with a role for pectin-related increased bundling of CMFs in this line. Stiffer cell walls in *OE CALPAIN*, with reduced levels of partially methyl-esterified HG and increased levels of highly methyl-esterified HG requires further investigation. The relationship between the degree of HG methyl-esterification and cell wall stiffness is unresolved. It could depend upon local concentrations of Ca^2+^ in the apoplast (Wang et al., 2020), orientation of the cell wall, such as anticlinal *vs* periclinal cell walls (Haas et al., 2020), activity of polygalacturonases (Yang et al., 2018) and many as yet unknown factors. Changes in interactions between pectins and CMF could also influence mechanical properties of the cell wall in *DEK1* modulated lines (**Figure 4E**). NMR studies of ‘never dried’ onion epidermal cell walls have shown that HGs make the majority of contacts with CMFs, which could mean that they act as primary mechanical tethers (Wang et al., 2015). Pectins affect cell wall mechanical properties through poro-elastic effects, without impacting the structural properties of cellulose (Lopez-Sanchez et al., 2015).

In future it will be essential to identify the proteolytic targets of DEK1 CALPAIN and the dynamics of cell wall changes in response to altered DEK1 to determine direct vs indirect effects. In this study, pectin composition and root inhibition by isoxaben have opposite patterns in *OE CALPAIN* and *dek1-4* lines, whereas cellulose composition and cell wall stiffness show similar trends. Further studies are required to determine if, for example, the CALPAIN domain is required for the former effects, whereas the latter effects are a response to the induced cell wall modifications. Different stress conditions are known to lead to similar responses of plants in terms of growth and morphogenesis (Tenhaken, 2014). However, the underlying molecular mechanism of these changes and their associated signaling cascades can differ greatly. CWI sensors of the CrRLK family, including FER and THE1 are involved in responses to stress, mediated by RALF peptide binding (Feng et al., 2018; Blackburn et al., 2020; Zhang et al., 2020). RALF peptides are known to be processed by substilin-like proteases (Stegmann et al., 2017), however, other proteases such as DEK1 may be involved in generating mature RALF ligands. DEK1 signaling cascades are still completely unknown and further work is required to determine if cross-talk between CrRLK signaling and DEK1 pathways occur. Identification of CALPAIN targets through the use of targeted proteomics approaches is essential for understanding the pathways through which DEK1 acts, and its precise role in plant growth and development.

## Material and methods

### Plant resources

*Arabidopsis thaliana* Columbia-0 (Col-0) ecotype was used as wild type (WT) control. *Dek1-4* is an EMS single point mutant (Roeder et al., 2012) backcrossed into Col-0 as described in (Galletti et al., 2015). The CALPAIN overexpression construct *pRPS5A*:*CALPAIN:6X HISTIDINE* (*OE CALPAIN*), with hygromycin resistance in Col-0 wild type background is described in Galletti et al., 2015. For CSC dynamics analysis we used *OE CALPAIN* and *dek1-4* crossed into CSC reporter line *pCESA3:GFP : CESA3* (Desprez et al., 2007).

Plants were grown vertically in petri dishes, on MS 1% (w/w) sucrose media, 0.8% agar, pH 5.8 (Murashige and Skoog, 1962), without vitamins. Plants were grown in Percival growth cabinets, model CU36L/LT (Percival Scientific, Perry, Iowa, USA), at a temperature of 21°C and light intensity of 95-111 mol m^–2^ s^–1^. Growth conditions used in different experiments are detailed in sections below.

F2 *DEK1* modulated lines crossed with GFP : CESA3 were used for CESA dynamics experiments. Non-genotyped segregating F2 plants were grown on MS 1% sucrose media for 3 days, imaged and then genotyped for identification of *DEK1* modulated lines containing *pCESA3:GFP : CESA3* constructs. After confocal imaging, *OE CALPAIN pCESA3:GFP : CESA3* F2 plants were transferred onto MS 1% sucrose media supplemented with 10 µg ml^-1^ hygromycin. Plants were grown horizontally for 10-14 days together with Col-0 and *GFP CESA3* plants as positive controls. Only imaging data for *OE CALPAIN pCESA3:GFP : CESA3* plants which survived the hygromycin selection were used for CESA dynamics analysis.

After CESA imaging, *dek1-4 pCESA3:GFP : CESA3* plants were transferred to MS 1% sucrose media supplemented with Plant Preservative Mixture (Plant Cell Technology, Washington DC, USA) diluted in ratio 1:1000. Plants were grown until they developed the first true leaves (7-10 days post imaging). A leaf was used to isolate DNA using Edwards buffer method (Edwards et al., 1991) and the DNA analyzed by dCAPS (Neff et al., 1998) (**Supplementary Table 2**) or KASP genotyping (LGC Genomics, Berlin, Germany) to identify *dek1-4* homozygous plants. Only imaging data for *dek1-4* homozygous plants was used in further CESA dynamics analysis.

### Analysis of *CESA* transcript levels

RNA was isolated from 6-day old *Arabidopsis* plants using the QIAGEN Rneasy Mini Kit (ID: 74104, QIAGEN, Hilden, Germany). RNA quality was determined using a Nanodrop 2000/2000c spectrophotometer (Thermo Fisher Scientific, Waltham, MA, USA). For relative quantification (RT)-qPCR experiments, cDNA synthesis was performed using Maxima First Strand cDNA Synthesis Kit (K1641, Thermo Fisher Scientific, Waltham, MA, USA) using 1 μg of RNA. Power SYBR Green Mastermix (4367659, Thermo Fisher Scientific, Waltham, MA, USA) was used for RT-qPCR and runs performed in 384 well plates (AB3384, Thermo Fisher Scientific, Waltham, MA, USA) in 5 μl reactions. Primers used for RT-qPCR are outlined in **Supplementary Table 2**. Lightcycler ABI 7900 HT (Applied Biosystems, Foster City, California, United States) lightcycler was used for RT-qPCR using amplification, conditions: 50° C – 2 min; 95° C –10 min; 40 cycles of 95° C for 15 s, 60° C for 1 min; 95° C for 15 s, 60° C for 15 s, 95° for 15 s. qPCR data was analyzed using SDS v 2.4.1 analysis software (Thermo Fisher Scientific, Waltham, MA, USA). *CESA* expression levels were normalized against *GLYCERALDEHYDE-3-PHOSPHATE DEHYDROGENASE* (*GADPH*) which was used as a reference gene.

Relative gene expression values expressed as fold changes were calculated using the method of (Livak and Schmittgen, 2001). *CESA* expression levels were treated as 1 in Col-0 WT and values in *DEK1* modulated lines were calculated compared to Col-0. Gene expression fold changes lower than 0.5 and higher than 2.5, compared to Col-0, were treated as significant.

### Cell wall composition analysis

Cell wall composition analysis was performed on pooled 10-day old whole seedlings. Analysis of the cell wall polysaccharide composition of alcohol insoluble cell wall residues (AIR) from Col-0 and *DEK1* modulated lines was performed according to (Pettolino et al., 2012). Analysis of the total crystalline cellulose in seedlings was performed using Updegraff assay (Updegraff, 1969) of AIR (10-50 mg).

### Immunofluorescence labelling

Cotyledon tissues from 10 day old seedlings were fixed and embedded according to (Wilson and Bacic, 2012). A Leica Ultracut R microtome (Leica Microsystems, Germany) was used to obtain 250 nm thin sections and labelled with JIM5 [low methyl esterified HG; (Knox et al., 1990)], JIM7 [high methyl esterified HG; (Knox et al., 1990)], and LM15 [xyloglucan; (Marcus et al., 2008)] antibodies at 1:10 dilution, secondary antibody was Alexa Fluor 488 goat anti-rat IgG (H+L) (Life Technology; # A48262) with 1:100 dilutions. For crystalline cellulose labelling, CBM3a (Blake et al., 2006) was used at 1:50 dilution, the secondary antibody used was anti-6x-His tag monoclonal (Invitrogen, # MA1-21315) with 1:100 dilutions, the third antibody was Alexa Fluor 488 goat anti-mouse IgG (H+L) (Life Technology; # A11006) with 1:100 dilutions. Images were acquired with an Olympus BX53 microscope under GFP channel. Calcofluor white (0.02%) was used to stain cellulose and images were acquired under UV channel. Two biological replicates from two independent lines were performed.

### Transmission-electron microscopy and immunolabelling

Thin sections (~80 nm) from cotyledon tissues were acquired as stated above. Antibody labelling and post-staining were performed according to (Wilson and Bacic, 2012). For pectin labelling, JIM5 and JIM7 antibodies were used at 1:15 dilution, secondary antibodies used was goat anti-rat 18 nm gold conjugated secondary antibody (Jackson Immuno Research #112-215-167) at 1:20 dilutions. For crystalline cellulose labelling, CBM3a at 1:50 dilution, secondary anti-6x-His tag monoclonal antibody (Invitrogen, # MA1-21315) at 1:175 dilutions, and the third antibody was goat anti-mouse 12 nm gold conjugated secondary antibody (Jackson Immuno Research #115-205-166) at 1:35 dilution. Grids were post-stained using 2% uranyl acetate for 10 min and Reynold’s lead citrate for 1 min. Grids were imaged using a Jeol 2100 EM equipped with a Gatan Orius SC 200 CCD camera. Two biological replicates from two independent lines were performed.

### Spinning disc confocal microscopy of CSC

Three-day old seedlings were mounted on microscope object slides and imaged using instruments and settings as described in (Sampathkumar et al., 2013). Photobleaching used to access CSC trafficking dynamics was performed using a FRAP/PA system (Roper Scientific, Acton, MA, USA) integrated into the spinning disc confocal imaging system, as described in (Sampathkumar et al., 2013).

### CSC timelapse processing

Image processing was performed using ImageJ software (Rasband, W., NIH, Bethesda, MD, USA). Brightness and contrast of the raw time-lapses were modified. Subtract background function (rolling ball size 30-40 pixels) and Walking average plugin (three frames averaged) were used to remove background noise and to obtain clearer images.

Density of CSC particles was determined from confocal time-lapse images using IMARIS 7.4 image analysis software (Bitplane, Oxford Instruments, Oxford, United Kingdom). Time-lapse image stacks were pre-processed in ImageJ as described above and imported into IMARIS. Spot detection and tracking function of the IMARIS was used to detect and label CSC particles. CSC particle numbers used to calculate mean CSC density were taken from three random timeframes from each analyzed pavement cell form every imaged cotyledon for each of the analyzed genotypes. Density was calculated as mean of CSC particles density per one μm^2^ for each cell, plant and genotype.

Velocity analysis of CESA particles imaged using confocal microscopy was performed using Fluorescent Image Evaluation Software for Tracking and Analysis (FIESTA) software (Ruhnow et al., 2011), which is available under open license from FIESTA, University of Dresden Fusion Forge Wiki webpage (https://fusionforge.zih.tu-dresden.de/plugins/mediawiki/wiki/fiesta/index.php/FIESTA). Raw confocal time-lapse images were imported into FIESTA. Maximum signal intensity projections were used to generate kymographs. Kymographs were generated from the CSC trajectories manually drawn using segmented line tool in FIESTA.

Analysis of the data obtained by FRAP was performed using a modified version of the CSC density measurement method described above. CSC particles detection and quantification was performed on 9 x 9 μm cell patches rather than 10 x 10 μm, which were the dimensions of bleached surface. After performing detection of fluorescently labelled CSC particles using IMARIS software, CSC post-FRAP density, recovery rates and average plasma membrane life-time of the complexes was calculated as described in (Sampathkumar et al., 2013).

### Cell wall isolation for AFM topographical imaging and nanoindentation

Cotyledon pavement epidermal cell walls were isolated for analysis with the atomic force microscope (imaging and nanoindentation). Rectangular microscope coverslips (Roth, Karlsruhe, Germany) were covered with 200 μl of 0.01% poly-D-lysine-hydrobromide (PDL; P6407, Sigma Aldrich, St. Luis, MO, USA) in deionized water. Coverslips were left for 15-20 min to allow the PDL to polymerize, after which coverslips were washed thoroughly with deionized water and dried in a stream of nitrogen.

We used 10-day old seedlings of *Arabidopsis* to obtain cotyledons for cell wall isolation. The larger of the two cotyledons was cut off using surgical tweezers, placed on a PDL coated coverslip (with the abaxial side facing the coverslip) and the following steps were performed under a stereomicroscope (Zeiss Stemi 508, Zeiss, Jena, Germany). The cotyledon was pressed gently on one side using surgical tweezers and then a vertical cut was made using a micro-surgery scalpel (Fine Scientific Tools, Vancouver, Canada), across the middle of the cotyledon in a direction perpendicular to its longer axis. After the vertical cut, a second cut was performed in parallel to the longer cotyledon axis to remove all leaf tissues and expose the abaxial epidermis with patches of the cell wall monolayers on its edge (**Supplementary Figure 6**). Regions with cell wall monolayers were observable under 50x magnification as small opaque patches of material on the edge of the abaxial epidermis (**Supplementary Figure 6**).

The remaining parts of the dissected cotyledon were carefully attached to the surface with nitrocellulose based red stained adhesive (nail polish) to immobilize the sample during imaging and mechanical probing. Adhesive was applied with an injection syringe needle, carefully avoiding regions with cell wall monolayers and left to cure for 5-10 seconds. Samples were then incubated with 500 μl of 1% SDS for 20-30 s to remove organelles, proteins and other cellular components released during the cotyledon dissection. SDS was removed by washing with deionized water, ensuring that the dissected cotyledon does not curl up on the coverslip. For high-resolution AFM topological imaging the samples were left overnight to completely dry while, samples used for mechanical measurements were stored in water to prevent loss of native mechanical properties.

### AFM topographical imaging

For measuring the CMF diameter, a Dimension 3100 AFM (Veeco, Plainview, NY, USA) was used. Imaging was performed in tapping mode in air, using silicon ARROW-NCR cantilevers (tip radius <10 nm; Nanoworld, Neuchâtel, Switzerland).The optical camera integrated into the AFM setup was used to identify regions of interest for imaging. Regions with a size of 2 x 2 μm^2^ were imaged with a resolution of 512 x 512 pixels. We recorded height channel images (**Supplementary Figure 8**) and amplitude channel images (**Figures 4A–C**). Imaging of all genotypes was performed in biological duplicate; that is, the same experiments were repeated twice with two sets of plants grown independently at different times.

### Analysis of the cellulose microfibril images

High-resolution, high-quality cell wall monolayers imaged in dry conditions were analyzed using custom made MATLAB script to determine the diameter of the imaged CMFs. For the analysis of CMF diameter, we used the height channel AFM images (**Supplementary Figure 8**) as obtained from the AFM software. The extracted values of the CMF diameter were pooled for each genotype and CMF diameter values.

### Mechanical characterization of isolated cell walls using atomic force microscopy

To determine the apparent elastic modulus of the isolated cell wall monolayers, we performed AFM-based nanoindentation (manual force mapping mode), applying a method previously described (Yakubov et al., 2016) and modified for our experimental conditions. Nanoindentation experiments were performed using a JPK Nanowizard 3 AFM (Bruker Nano GmbH, Berlin, Germany) mounted on an Olympus IX71 inverted phase-contrast microscope (Olympus Corporation, Shinjuku City, Tokyo, Japan). Coverslips with prepared samples were mounted onto the AFM sample holder as described in (Yakubov et al., 2016).

An optical CCD camera integrated into the AFM setup was used during the entire measurement; first to align the cantilever and then to perform constant checks of the sample and cantilever properties. We used DNP-10 cantilevers (cantilever A) with a nominal spring constant of 0.35 N m^-1^ (Bruker Corporation, Billerica, MA, USA). The optical sensitivity of the cantilevers was determined from contactless oscillations after which the thermal noise method was used to obtain the spring constant. The used cantilevers had spring constants ranging from 0.30-0.38 N m^-1^.

Before starting the mechanical characterization, topological imaging of the cell wall monolayer was performed to identify the thinnest regions of the sample. First, using intermittent contact mode, we imaged an area of 5 x 5 μm^2^ with a resolution of 512 x 512 pixels. Imaging of a 5 x 5 μm^2^ region was always performed near the edge of the dissected cotyledon, so that part of imaged area represented the bare glass slide to which the cotyledon was attached. The flat glass slide surface, which contained some tissue debris after dissection, was used as a reference (position 0 nm), to determine the thickness of the imaged sample (**Supplementary Figure 7**). If sample thickness was in the 0.5-2.0 μm range, we selected a smaller 1 x 1 μm^2^ region within the original 5 x 5 μm area and repeated the topography imaging to re-confirm cell wall thickness (**Supplementary Figure 7**). If the thickness of the topological features inside this region remained in the 0.5-2 μm range, we performed nanoindentation on that region. This range of sample thicknesses values was empirically chosen. During method development, we determined that regions thicker than 2.0 μm were either wrinkled cell wall patches or cell wall bilayers (i.e. from both adaxial and abaxial sides; thickness 2.5-4 μm). Samples thinner than 0.5 μm were not used to avoid non-specific surface interactions and to minimize the contribution of the underlying glass surface to the elastic modulus.

For nanoindentation, the 1 x 1 μm^2^ region of interest was further divided into a 16 x 16 array. Each position was indented once to obtain a force map consisting of 256 force-displacement curves. For each approach-retract cycle, the contact force was set to 2.5 nN and the z-piezo speed was 1 μm s^-1^. The average indentation depth was 180 nm for Col-0, 164 nm for *OE CALPAIN* and 147 nm for *dek1-4*, while indentation depth values where mostly in the range of 100-350 nm (**Supplementary Figure 9G**). While the indentation depths partially exceeded 10% of the sample thickness, the values lie in a similar range. Contributions from the underlying glass surface can thus be expected to be similar for all three samples.

One force map was obtained from each dissected cotyledon. Imaging of all genotypes was performed in biological duplicate; the same experiments were repeated two times, with two sets of plants grown independently at different times. From each replicate, cotyledons from 4-6 different plants were imaged, one cotyledon per plant. Force maps were analyzed using JPK SPM processing software, version 6.1.9 (Bruker Nano GmbH). As adhesion was observed in the retract segments of the force-displacement curves, the approach segments were fit with the Hertz model to obtain the apparent elastic modulus. The Poisson ratio was set to 0.5 and the baseline value was unpinned and set to 0. The extracted apparent elastic moduli from different cotyledons for the same genotype were pooled and *dek1-4* and *OE CALPAIN* values were compared with Col-0.

### Isoxaben and salt assays

Plants used for the isoxaben treatments were sterilized and grown for 6 days on MS media containing 1% sucrose, with the addition of 2 nM isoxaben (Sigma-Aldrich, St. Louis, MO, USA) dissolved in DMSO (Honeywell, Charlotte, NC, USA), and on MS 1% sucrose containing media supplemented with 2nM DMSO as a control. Experiments were performed in biological duplicates and technical triplicates. Salt stress experiments were performed as described in (Feng et al., 2018).

Plants were imaged using a Keyence VHX-6000 digital microscope (Keyence Corporation of America, Itasca, IL, USA). Whole plates were placed on a motorized microscope stage and series of images were acquired in sequence covering the entire surface of the plate, and then automatically stitched by the software to generate a single whole-plate image. We used objective ZS20, 20X magnification, on black field, with epi-illumination, for imaging 6-day isoxaben grown and salt-treated plants. Root lengths were quantified using Measure length function of ImageJ software (Rasband, W., NIH, Bethesda, MD, USA).

### Statistical analysis

Unless stated otherwise, all statistical analysis in this study were performed using unpaired Student t-test, results were treated statistically significant if *p*<0.05. We used online Student t-test calculator at the webpage https://www.graphpad.com/quickcalcs/ttest1.cfm.

Analysis of data from isoxaben growth and salt assays was performed using estimation statistics. After obtaining root length data, statistical significance of the results was assessed using the online, open-access Estimation Stats software (https://www.estimationstats.com/#/) and data are plotted using Cumming estimation plot as described in (Ho et al., 2019).

## Data availability statement

The original contributions presented in the study are included in the article/**Supplementary Material**. Further inquiries can be directed to the corresponding authors.

## Author contributions

LN performed CSC imaging, AFM measurements and imaging and CWI sensing experiments, and analyzed all the data from these experiments. KJ, TB, and AS conceptualized and designed the research. LN, GY, KJ, and KB conceptualized, developed, and validated cell wall isolation method and AFM imaging and force spectroscopy protocols. KJ and AS made *DEK1* crosses with CSC reporter line. YM performed immunofluorescences and immunohistochemical imaging experiments and analyzed the data. GY wrote the MATLAB script for analysis of AFM cellulose images. LN wrote the original draft of the article manuscript. KJ, TB, KB, and AS edited, curated and corrected the original draft of the manuscript. KB, GY, and YM gave valuable input and comments on the article manuscript.
