# Supplementary material for: DEFECTIVE KERNEL1 regulates cellulose synthesis and affects primary cell wall mechanics
Source: Front Plant Sci. 2023 Mar 14;14:1150202. doi: 10.3389/fpls.2023.1150202 (PMC10043484; doi:10.3389/fpls.2023.1150202)
Supplement: Supplementary file 1 [file DataSheet_1.pdf]

Supplementary Material

DEFECTIVE KERNEL1 (DEK1) regulates cellulose synthesis and affects primary cell wall mechanics

Lazar Novaković, Gleb E. Yakubov, Yingxuan Ma, Antony Bacic, Kerstin G. Blank, Arun Sampathkumar, Kim L. Johnson*.

*** Correspondence:** Corresponding Author: k.johnson@latrobe.edu.au

SUPPLEMENTARY TABLES

Supplementary Table 1. Linkage data from methylation analyses derived from alcohol insoluble residues of WT, *OE CALPAIN* and *dek1-4* 10-day old seedlings from two biological replicates and average of technical duplicates.

		WT-1	WT-2	*OE CALPAIN-1*	*OE CALAPIN-2*	*dek 1-4-1*	*dek1-4-2*
**Pectic Polysaccharides**							
**Arabinan**	1,5-Ara (f)	2.54	3.78	3.42	5.12	2.05	3.82
	1,3,5-Ara (f)	0.38	0.61	0.53	1.15	0.32	0.74
	1,2,5-Ara (f)	0.21	0.48	0.37	0.65	0.21	0.56
	t-Ara	0.59	1.09	0.9	1.8	0.54	1.3
	**Total Arabinan**	**3.73**	**5.97**	**5.22**	**8.72**	**3.12**	**6.42**
**Type I AG**	1,4-Gal (p)	2.07	2.72	2.83	3.83	2.39	2.52
	1,3,4-Gal (p)	0.55	0.52	0.64	1.14	0.63	0.64
	1,4,6-Gal (p)	0.17	0.13	0.2	0.33	0.2	0.19
	t-Ara	0.72	0.65	0.84	1.47	0.83	0.83
	**Total Type I AG**	**3.51**	**4.02**	**4.51**	**6.78**	**4.05**	**4.18**
**Type II AG**	1,3-Gal (p)	0.55	0.13	0.44	0.22	0.31	0.2
	1,6-Gal (p)	0.64	0.7	0.93	1.01	0.56	1.04
	1,3,6-Gal (p)	0.83	0.95	1.22	1.35	0.81	1.36
	t-Ara	0.83	0.95	1.22	1.35	0.81	1.36
	**Total Type II AG**	**2.85**	**2.73**	**3.81**	**3.92**	**2.49**	**3.97**
**HG**	1,4-Gal A	0.05	0.38	0.2	0.62	0.2	0.67
	/1,4-GalA (OMe)	/0.35	/0.77	/0.79	/1.02	/0.38	/1.04
	t-Gal A	0.11	0.29	0.4	0.29	0.07	0.26
	**Total HG**	**0.51**	**1.44**	**1.39**	**1.92**	**0.65**	**1.97**
**RG I**	1,4-Gal A	0.18	0.41	0.37	0.67	0.14	0.56
	1,2-Rha (p)	0.09	0.02	0.22	0.08	0.09	0.06
	1,2,4-Rha (p)	0.1	0.39	0.15	0.59	0.06	0.5
	**Total RG I/II**	**0.37**	**0.82**	**0.73**	**1.34**	**0.29**	**1.11**
**Glucuronoxylan**	1,4-Xyl (p)	2.23	3.47	4.63	4.39	2.83	3.66
	1,2,4-Xyl (p)	0.2	0.73	0.28	1.12	0.34	0.83
	t-Glc A	0.12	0.15	0.15	0.31	0.12	0.26
	**Total Glucuronoxylan**	**2.54**	**4.36**	**5.06**	**5.82**	**3.3**	**4.75**
**Heteromannan**	1,4-Man (p)	2.24	2.8	2.24	2.64	1.83	2.69
	1,4,6-Man (p)	0.33	0.77	0.38	1.05	0.35	0.83
	1,4-Glc (p)	2.24	2.8	2.24	2.64	1.83	2.69
	1,4,6-Glc (p)	0.33	0.77	0.38	1.05	0.35	0.83
	t-Gal	0.66	1.54	0.75	2.11	0.7	1.66
	**Total Heteromannan**	**5.8**	**8.68**	**5.99**	**9.49**	**5.07**	**8.7**
**Xyloglucan**	1,4,6-Glc (p)	3.77	4.82	2.74	4.41	3.52	5.05
	1,4-Glc (p)	3.77	4.82	2.74	4.41	3.52	5.05
	1,2-Xyl (p)	0.97	1.52	0.53	1.54	1.38	1.39
	1,2-Gal (p)	0.41	1.23	0.22	1.11	0.29	1.1
	t-Fuc (p)	0.41	1.06	0.22	1.03	0.29	1.05
	t-Xyl	1.5	2.71	1.68	2.67	1.67	3.43
	t-Gal	0.97	1.06	0.53	0.71	1.38	0.57
	**Total Xyloglucan**	**11.8**	**17.23**	**8.66**	**15.89**	**12.03**	**17.63**
**Cellulose**	1,4-Glc	65.24	47.45	57.93	36.37	62.49	43.75
	**Total Cellulose**	**65.24**	**47.45**	**57.93**	**36.37**	**62.49**	**43.75**
**Callose**	1,3-Glc (p)	0.08	0.32	0.29	0.48	0.06	0.32
	**Total Callose**	**0.08**	**0.32**	**0.29**	**0.48**	**0.06**	**0.32**
**Extensin**	1,2-Ara (f)	0.9	1.67	1.27	1.4	1.13	1.53
	1,3-Ara (f)	0.34	0.63	0.48	0.53	0.42	0.57
	t-Ara (f)	0.45	0.84	0.64	0.7	0.57	0.77
	t-Gal	0.01	0.21	0.16	0.18	0.14	0.19
	**Total Extensin**	**1.7**	**3.34**	**2.55**	**2.8**	**2.26**	**3.06**
	**Total**	**98.11**	**96.35**	**96.15**	**93.54**	**95.8**	**95.88**

Supplementary Table 2. List of PCR primers used in this study

Gene	Primer name	Primer sequence	Application	Reference
AT1G55350	*dek1-4*	FW: 5’-TGTTGGTGGAACAGACTATGTGAATTCA-3’	dCAPS genotyping	Roeder et al., 2012
		RV: 5’-TGAAGACTGAAAGGACAAAAGGTGC-3’		
AT4G32410	*CESA1*	FW: 5’-GATCCGACATGAATCTGATGG-3’	RT-qPCR	Chaudhary et al., 2020
		RV 5’-CACATTCATTACACGCGACA-3’		
AT5G05170	*CESA3*	FW: 5’-GTCAGATTGGGGAATGGAGA-3’	RT-qPCR	Chaudhary et al., 2020
		RV 5’-GCTTAGGCATGCAGTAAATGG-3’		
AT5G64740	*CESA6*	FW: 5’-ACCCGGATTTGATCACCATA-3’	RT-qPCR	Chaudhary et al., 2020
		RV 5’-GAACCCCAGAGACTCGTATCA-3’		
AT3G04120	*GADPH*	FW: 5’-TTGGTGACAACAGGTCAAGCA-3’	RT-qPCR	Czechowski et al. 2005
		RV 5’-AAACTTGTCGCTCAATGCAATC-3’		

SUPPLEMENTARY FIGURES

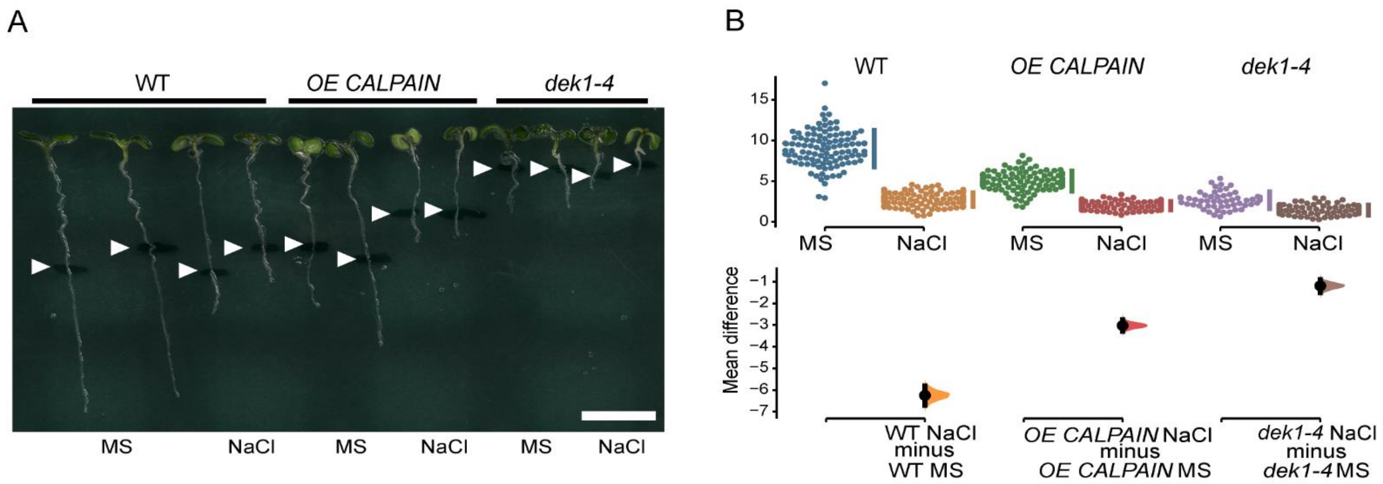

Supplementary Figure 1. Response of WT and *DEK1* modulated *Arabidopsis* lines to salt stress. (A) Representative images of 7-day old WT, *OE CALPAIN* and *dek1-4* seedlings transferred for 48 h on media supplemented with or without 140 mM NaCl. White arrowheads indicate length of roots at transfer. Scale bar = 5 mm. (B). Mean difference for root length for each genotype between plants grown on control media and plants grown on NaCl supplemented media. The raw data are plotted on the upper axes; each mean difference is plotted on the lower axes as a bootstrap sampling distribution. Mean differences are depicted as dots on the lower panels of Cumming plots; 95% confidence intervals are indicated by the ends of the vertical error bars**.**N = 107 WT MS, 108 WT NaCl, 95 *OE CALPAIN* MS, 97 *OE CALPAIN* NaCl, 53 *dek1-4* MS, 76 *dek1-4* NaCl.

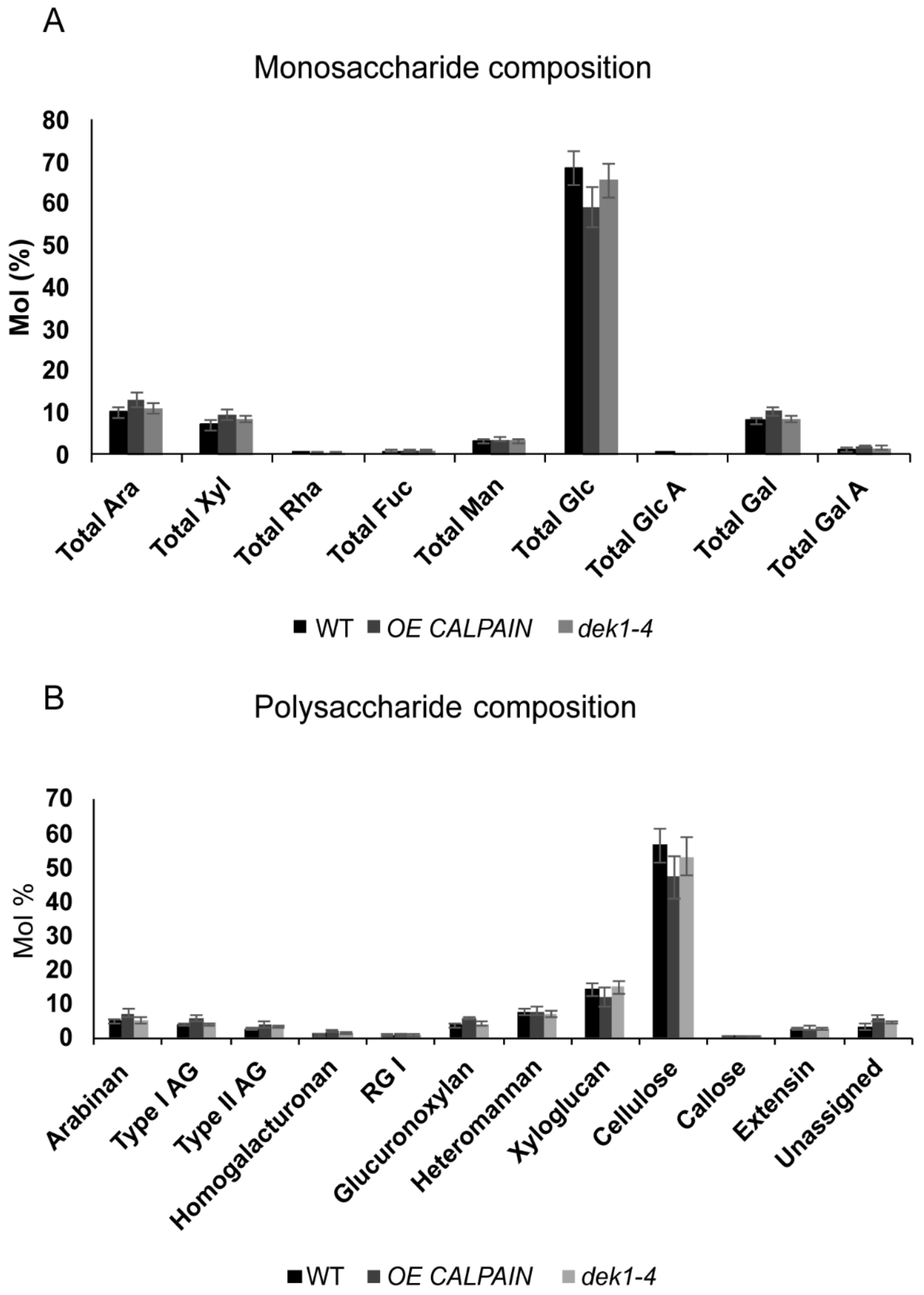

Supplementary Figure 2. Monosaccharide and polysaccharide composition of alcohol insoluble residue (AIR) cell wall preparations from 10-day old seedlings of WT and *DEK1* modulated lines. (**A**) Monosaccharides shown as Mol % of AIR. (**B**) Polysaccharide composition of WT and *DEK1* modulated lines. No statistical differences were observed. Ara: arabinose. Xyl: xylose. Rha: rhamnose. Fuc: fucose. Man: mannose. Glc: glucose. GlcA: glucuronic acid. Gal: galactose. GalA: galacturonic acid. AG: arabinogalactan. HG: homogalacturonan. RG: rhamnogalacturonan. Data shown are the average of two biological replicates with two technical replicates each. Error bars represent standard error. See Supplementary Table 1 for linkage data.

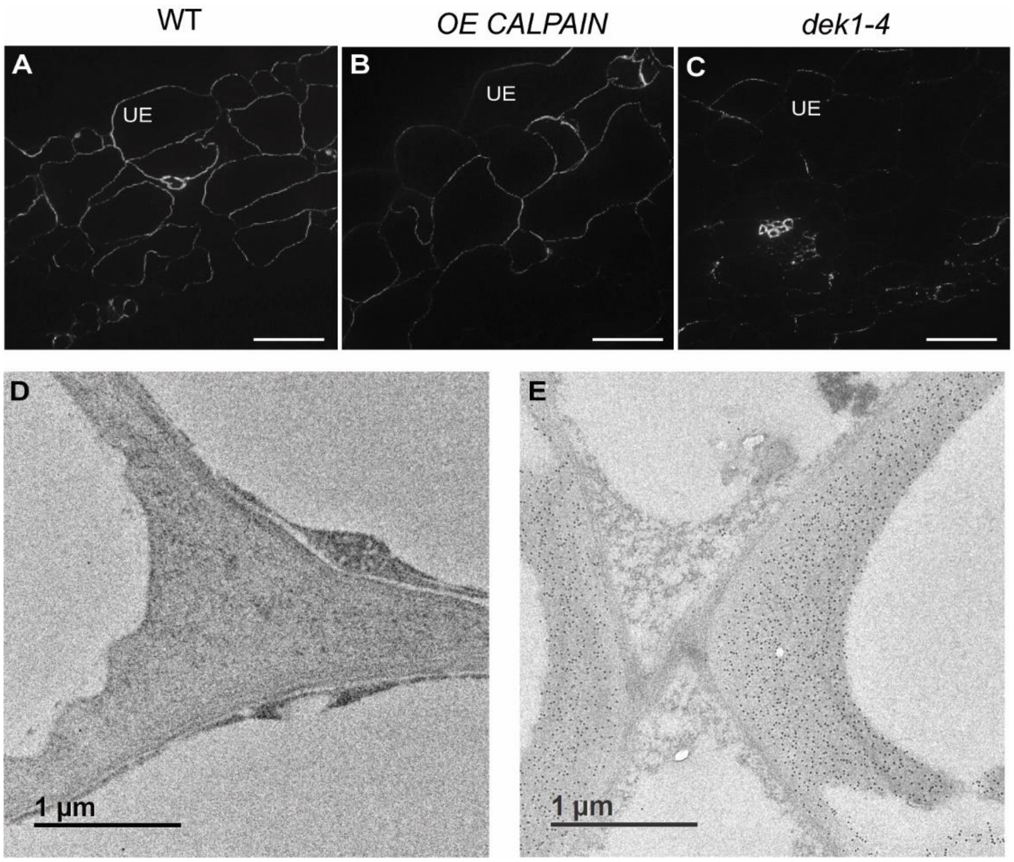

Supplementary Figure 3. Labelling of CBM3a cellulose binding protein. Immunofluorescence labelling of CBM3a in WT (**A**), *CALPAIN OE* (**B**) and *dek1-4* (**C**). UE = upper epidermis. **D-E**. CBM3a immuno-gold labelling visualised using transmission electron microscopy negative control with no CBM3a (**D**) and positive control (**E**) with extensive labelling of cellulose in xylem vessels of WT (see Figure 1). Scale bar in A-C = 20 μm and 1 μm in D,E.

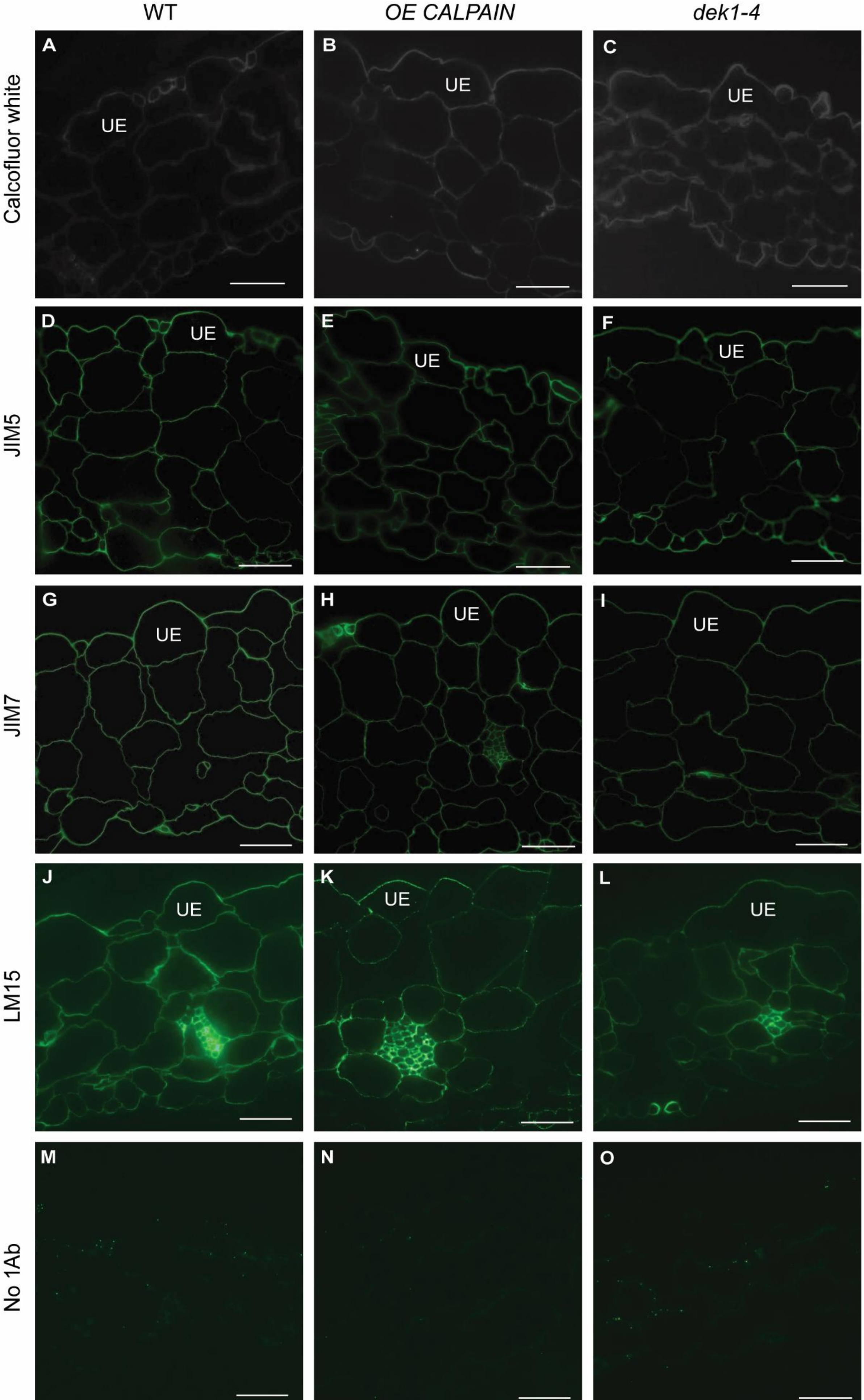

WT
OE CALPAIN
dek1-4
Calcofluor white
JIM5
JIM7
LM15
No 1Ab
A
B
C
D
E
F
G
H
I
J
K
L
M
N
O
UE

Supplementary Figure 4. Immunofluorescence labelling of selected antibodies and dyes detecting cell wall polysaccharides in WT and *DEK1* modulated lines. No visible differences in immunofluorescence intensity for Calcofluor white staining of cellulose (**A-C**), JIM5 (**D-G**) and JIM7 (**H-I**) antibodies for labelling of partial and highly methyl-esterified homogalacturonan pectin, respectively and LM15 (**J-L**) labelling for xyloglucan. (**M-O**) No primary antibody control. UE = upper epidermis. Scale bars = 20 µm.

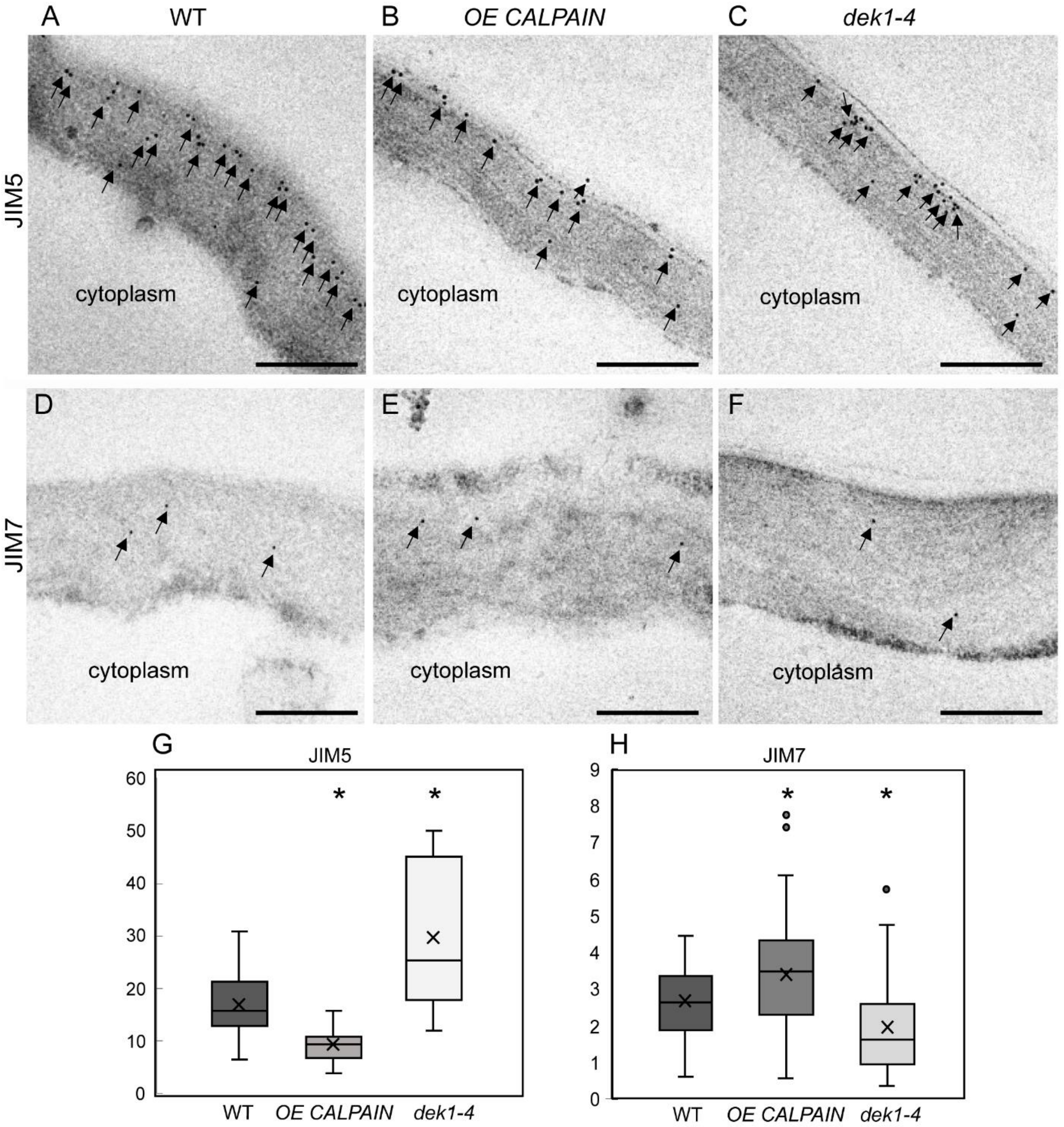

Supplementary Figure 5. Immunogold detection of pectin epitopes in outer epidermal cell walls of 10-day old cotyledons in WT and *DEK1* modulated lines. Transmission electron micrographs of epidermal cell walls labelled with JIM5 antibody, for partially methyl-esterified HG (A-C) and JIM7 antibody, for highly methyl-esterified HG (D-F). Black arrows (A-F) are pointing to the immunogold particles. (G) Average immunogold particles density for JIM5 antibody; WT: 18.86±0.82 μm^{-2}, *OE CALPAIN*: 15.79±1.10 μm^{-2}, *dek1-4*: 26.73±1.91 μm^{-2}. Asterisks denote statistical significance compared to WT, $p<0.0001$ for *dek1-4*, $p=0.02$ for *OE CALPAIN*, unpaired Student t-test. Experiment was performed in biological duplicates; n=75 cell walls for Col-0, 36 for *OE CALAPIN*, 30 for *dek1-4*. (H) Average immunogold particles density for JIM7 antibody; WT; 2.63±0.19 μm^{-2}, *OE CALPAIN*: 3.35±0.24 μm^{-2}, *dek1-4*: 1.90±0.21 μm^{-2}. Asterisks denote statistical significance,

p=0.03 for *OE CALPAIN*, *p*=0.0126 for *dek1-4*, unpaired Student t-test. Experiment was performed in biological duplicates; n=36 cell walls for Col-0, 50 for *OE CALAPIN*, 35 for *dek1-4*.

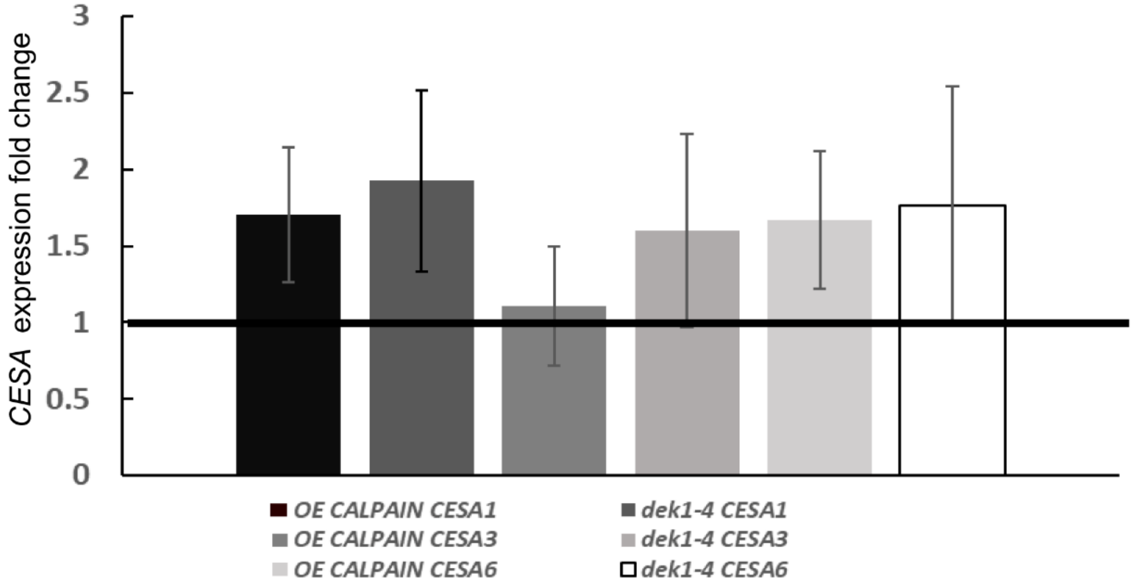

Supplementary Figure 6. RT-qPCR analysis of primary cell wall CESA transcript levels in 6-day old seedlings. CESA transcript levels analyzed by RT-qPCR and relative expression ΔΔCt method (Livak and Schmittgen, 2001). WT CESA levels were set as a reference point with a value of 1. DEK1 modulated lines showed no significant difference (fold change >2 or <0.5) in CESA expression. Data represent mean of three biological replicates and three technical replicates.

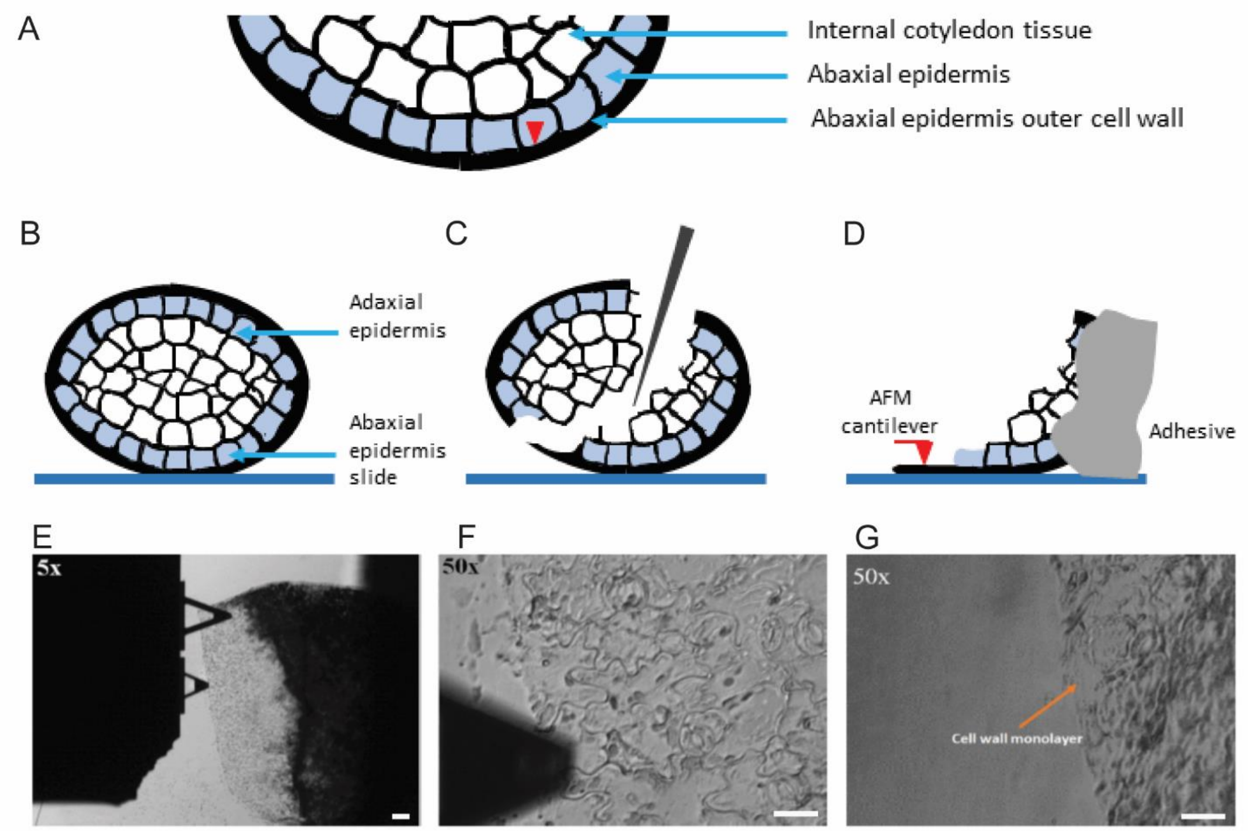

Supplementary Figure 7. Cotyledon dissection protocol developed for cell wall monolayer isolation. Schematic representation of an intact *Arabidopsis* cotyledon (**A, B**). Cotyledon was first dissected using a micro scalpel, with an incision perpendicular to its long axis. With this cut, the cotyledon was opened, and its internal tissues exposed (**C**). A second cut was made parallel to the cotyledon's longer axis resulting in removal of internal cotyledon tissues. Isolated abaxial epidermal cell wall monolayer was attached to a microscope coverslip coated with poly-D-lysine. Cotyledon was attached to the glass slide with an adhesive (nail polish), to immobilize it for AFM analysis (**D**). Dissected cotyledon with exposed abaxial epidermis with AFM probes DNP-10 A and B visible in vicinity of the sample. (E). Dissected cotyledon with abaxial epidermis cell wall monolayer exposed, ready for AFM assays (**F, G**). Scale bar = 100 µm (**E**), scale bar = 2 µm (**F,G**).

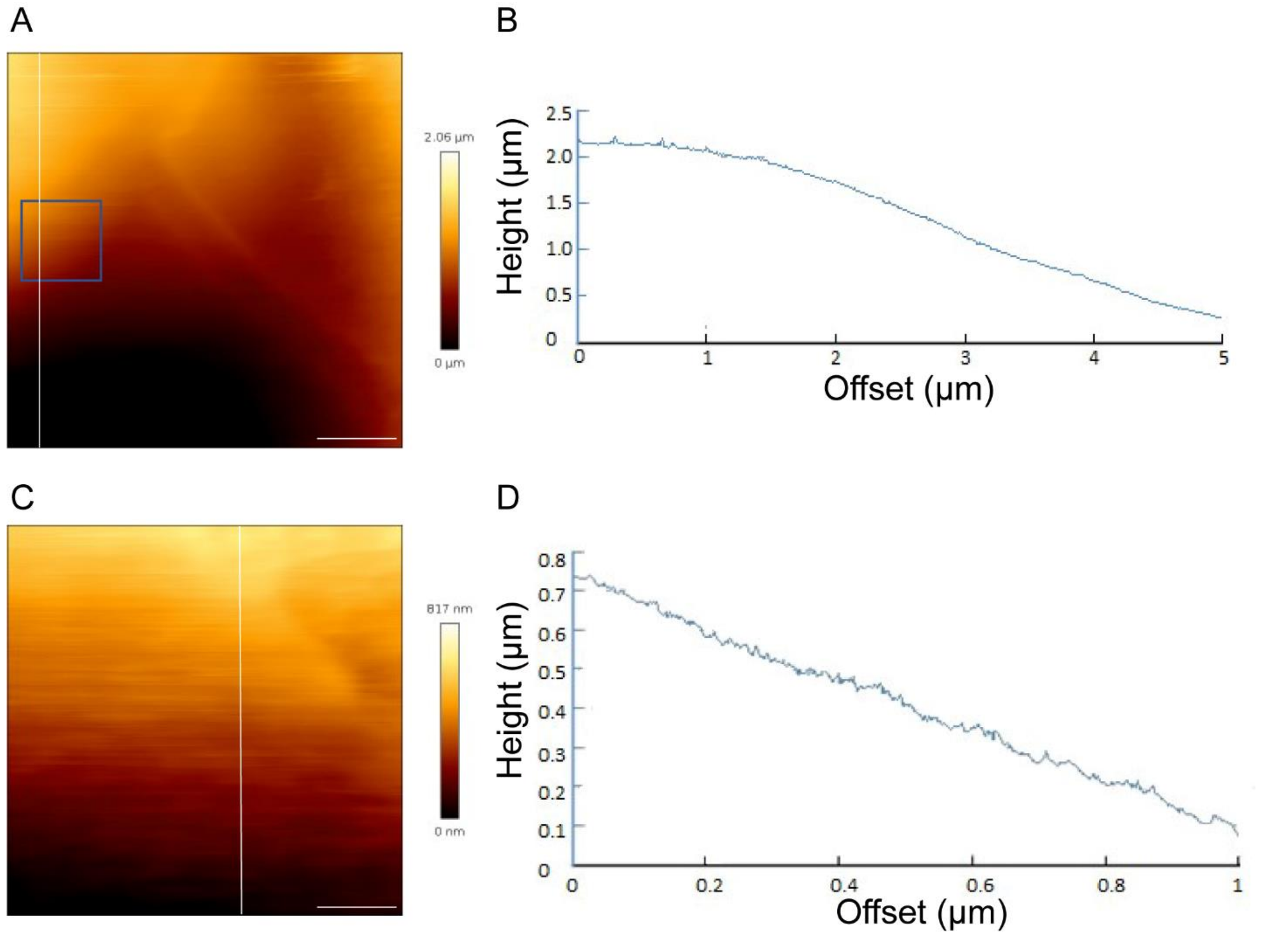

Supplementary Figure 8. Representative topographical AFM images of dissected cotyledons made to identify regions for mechanical characterization of the cell wall monolayer. Dissected cotyledons were imaged using intermittent contact mode, in water, using AFM Nanowizard 3 in order to identify cell wall monolayers which were then mechanically characterized. (**A**) A region of the cell wall (5 x 5 µm^2) was initially imaged on the edge of the dissected cotyledon to identify potential monolayer regions. Every scan included a small area of the glass slide onto which the cotyledon was placed so that the sample height could be determined with the glass slide as a reference. The black region on the 5 x 5 µm^2 image represents the surface of the glass slide. Scale bar = 1 µm. (**B**) Height profile along the white line in **A**. (**C**) If a region with a height of 0.5-2 µm was identified in the initial 5 x 5 µm^2 scan, a smaller region of interest (1 x 1 µm^2; blue square in **A**) was identifed and imaged again. Scale bar = 200 nm. (**D**) Height profile along the white line in **C**. In case that the topographical features in the 1 x 1 µm^2 region again displayed a height in the 0.5-2 µm range, force mapping was perfomed in the respective region in order to determine the apparent elastic moduli.

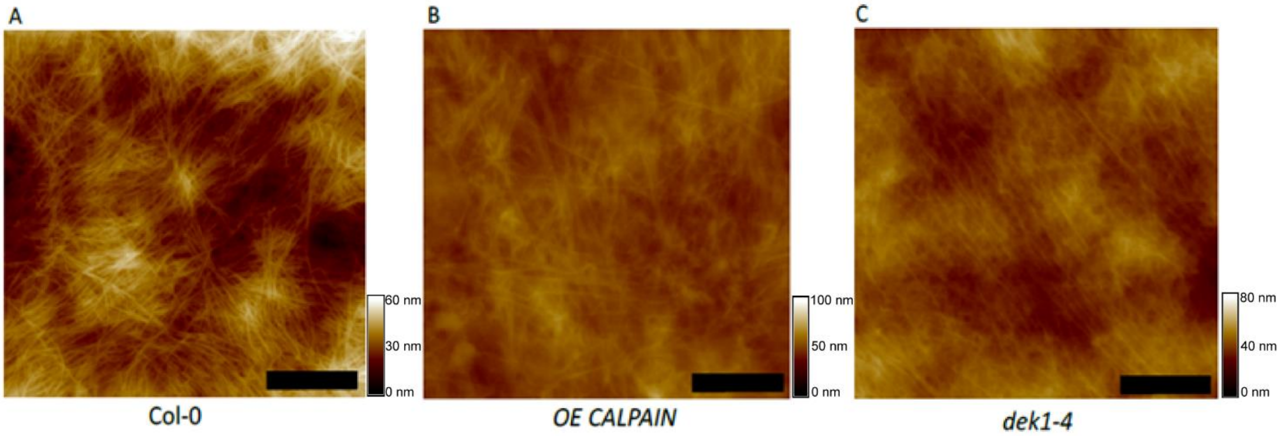

Supplementary Figure 9. AFM height signal images of epidermal cotyledon cell walls. Height channel images correspond to topographical features of the scanned cell wall monolayers. Raw height channel images were used for CMF bundle diameter analyses (Figure 4D and 4E). Scale bars = 250 nm.

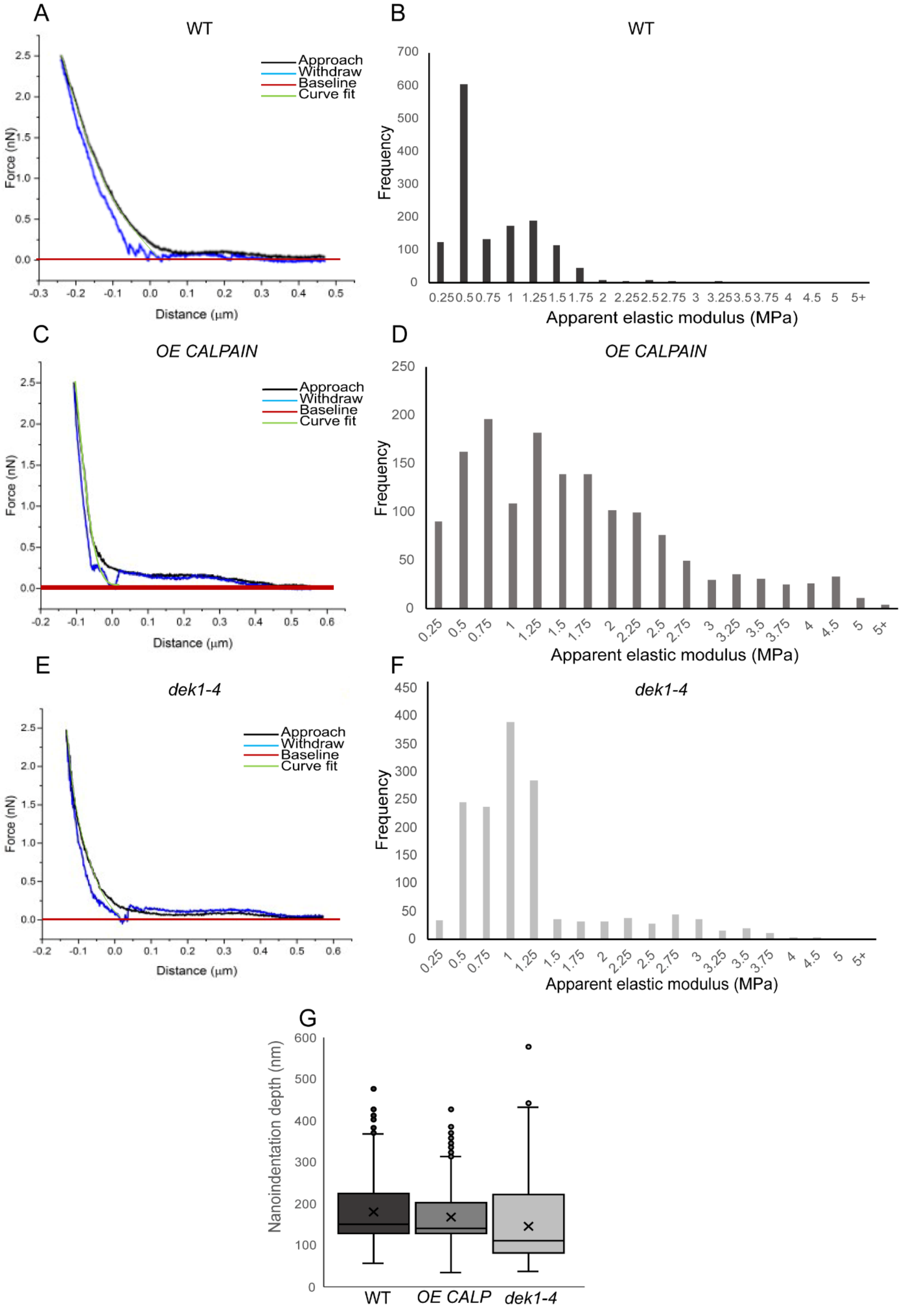

A
WT
Approach
Withdraw
Baseline
Curve fit
Force (nN)
Distance (µm)
B
WT
Frequency
Apparent elastic modulus (MPa)
C
OE CALPAIN
Approach
Withdraw
Baseline
Curve fit
Force (nN)
Distance (µm)
D
OE CALPAIN
Frequency
Apparent elastic modulus (MPa)
E
dek1-4
Approach
Withdraw
Baseline
Curve fit
Force (nN)
Distance (µm)
F
dek1-4
Frequency
Apparent elastic modulus (MPa)
G
Nanoindentation depth (nm)
WT
OE CALP
dek1-4

Supplementary Figure 10. Mechanical characterization of cotyledon epidermal cell wall monolayers. (**A,C,E**) Representative force-displacement curves obtained by AFM nanoindentation of the dissected cell wall monolayers. The approach segment was fit with the Hertz model. (**B,D,F**) Frequency distribution histograms showing the apparent elastic moduli for the three analyzed genotypes (pooled for all analyzed cotyledons). (**G**) Box-plot of cantilever indentation depths for all three genotypes (pooled for all analyzed samples).

References

Chaudhary, A., Chen, X., Gao, J., Leśniewska, B., Hammerl, R., Dawid, C., Schneitz, K. (2020) The Arabidopsis receptor kinase STRUBBELIG regulates the response to cellulose deficiency. PLOS Genetics, 16(1), e1008433. doi: 10.1371/journal.pgen.1008433

Czechowski, T., Stitt, M., Altmann, T., Udvardi, M. K., and Scheible, W.-R. (2005). Genome-wide identification and testing of superior reference genes for transcript normalization in arabidopsis. Plant Physiol. 139, 5–7. doi: 10.1104/pp.105.063743

Livak, K. J. & Schmittgen, T. D. 2001. Analysis of relative gene expression data using real-time quantitative PCR and the $2^{(-\Delta\Delta Ct)}$ Method. *Methods,* 25.

Roeder, A. H. K., Cunha, A., Ohno, C. K., and Meyerowitz, E. M. (2012). Cell cycle regulates cell type in the arabidopsis sepal. Development 139, 4416–4427. doi: 10.1242/dev.082925

frontiers
